# KSHV vIRF3–PKM2 interaction induces SMAD5 acetylation, promoting Kaposi’s sarcoma progression

**DOI:** 10.1038/s12276-026-01849-2

**Published:** 2026-10-05

**Authors:** Yeong Jun Kim, Juhyeon Lim, Jae Jin Lee, Abhishek Kumar, Shrute Kannappan, Junjie Zhang, Soochi Kim, Han-Jun Kim, Kyeong Kyu Kim, Zsolt Toth, Hyungjin Eoh, Kyung-Chul Choi, Hye-Ra Lee

**Affiliations:** 1 Department of Biotechnology and Bioinformatics, College of Science and Technology, Korea University, Sejong, Republic of Korea; 2 Department of Molecular Microbiology and Immunology, University of Southern California, Los Angeles, CA USA; 3 Department of Oral Biology, College of Dentistry, University of Florida, Gainesville, FL USA; 4 Department of Precision Medicine, Sungkyunkwan University School of Medicine, Suwon, Republic of Korea; 5 The State Key Laboratory of Virology, Medical Research Institute, Wuhan University, Wuhan, China; 6 College of Pharmacy, Korea University, Sejong, Republic of Korea; 7 Department of Ophthalmology, University of Southern California, Los Angeles, CA USA; 8 Department of Biochemistry and Molecular Biology, Brain Korea 21 Project, Asan Medical Center, University of Ulsan, College of Medicine, Seoul, Republic of Korea; 9 Department of Lab Medicine, College of Medicine, Korea University, Seoul, Republic of Korea

**Keywords:** Tumour virus infections, Metabolomics

## Abstract

Kaposi’s sarcoma (KS), induced by Kaposi’s sarcoma-associated herpesvirus (KSHV), is a multicentric tumor of lymphatic endothelial origin. However, the fundamental molecular mechanisms underlying the KS progression remain poorly understood. Here we found that interaction between KSHV vIRF3 and PKM2 controls the metabolic switch of lymphatic endothelial cells (LECs), inducing disseminated visceral KS in xenograft models of KSHV-infected LECs. Mechanistically, vIRF3–PKM2 interaction elevates intracellular acetyl coenzyme A (acetyl-CoA) level by augmenting the PKM2 enzymatic activity, leading to SMAD5 acetylation, which contributes to the endothelia-to-mesenchymal transition of LECs. Notably, a vIRF3-derived short peptide acts as a potent and selective PKM2 antagonist, effectively regressing KS progression. Collectively, these results suggest that vIRF3 plays a pivotal role in KS malignancy by linking acetyl-CoA metabolic reprogramming to cancer progression.

## vIRF3 and PKM2 link metabolism to Kaposi’s sarcoma progression

This study delves into the metabolic reprogramming associated with Kaposi’s sarcoma (KS), focusing on the role of acetyl coenzyme A (acetyl-CoA) and its synthesis pathways. Researchers identified a critical interaction between the Kaposi’s sarcoma-associated herpesvirus (KSHV) protein vIRF3 and the enzyme pyruvate kinase M2 (PKM2), which enhances PKM2 activity, leading to increased acetyl-CoA levels. This interaction promotes the tetramerization of PKM2, boosting pyruvate kinase activity and mitochondrial metabolism and suppressing fatty acid oxidation. Elevated acetyl-CoA levels result in increased SMAD5 acetylation, triggering endothelial-to-mesenchymal transition and enhancing KS progression. Importantly, a vIRF3-derived peptide, VDP, was found to inhibit this interaction, reducing tumor growth in a mouse model. This work highlights the vIRF3–PKM2–acetyl-CoA axis as a potential therapeutic target for KS.

This summary was initially drafted using artificial intelligence, then revised and fact-checked by the author.

## Introduction

Metabolic reprogramming is essential for the initiation and progression of cancer. Despite a strong reliance on glycolysis under aerobic conditions, most tumors retain mitochondrial respiration to fuel the tricarboxylic acid (TCA) cycle and support anabolic pathways^1^. This dual capacity not only challenges Warburg’s original hypothesis that defective respiration is essential for tumor initiation but also underscores metabolic plasticity as a hallmark of tumor progression^2,3^. Among the central hubs of this plasticity, acetyl coenzyme A (acetyl-CoA) serves as a key molecule in cell metabolism and in epigenetic and transcriptional regulation, having a crucial role in a wide range of cellular processes^4^. Acetyl-CoA links glycolysis to the TCA cycle by mediating the conversion of pyruvate, donates acetyl groups for protein acetylation, and functions as a critical precursor for key biosynthetic pathways, including lipid synthesis^5^. Dysregulation of acetyl-CoA levels contributes to critical features of tumorigenesis and may present novel opportunities for therapeutic intervention. Therefore, acetyl-CoA synthesis pathways, which include pyruvate catabolism during glycolysis, fatty acid β-oxidation (FAO), and amino acid catabolism, have been shown to be altered in a cancer-type-dependent manner^4,6^. Despite substantial advances in understanding the metabolic functions of acetyl-CoA in tumorigenesis, its role in regulatory mechanisms underlying Kaposi’s sarcoma (KS) progression remains largely undefined.

KS is a multifocal neovascular and lymphatic endothelial-derived tumor that is etiologically linked to Kaposi’s sarcoma-associated herpesvirus (KSHV)^7^. Histologically, KS is marked by aberrant angiogenesis, erythrocyte-rich vascular slits, and the presence of proliferating spindle cells infected with KSHV^8^. Clinically, it presents as multiple lesions involving the skin, oral, lungs, and gastrointestinal tract, often obscuring clonal relationships among lesions^9^. KS is subclassified into four clinical stages, ranging from indolent macular and nodular lesions (stage I) to disseminated mucocutaneous and visceral involvement (stage IV), the latter reflecting aggressive tumor biology^10–12^. This progression is considered to be driven, at least in part, by metabolic reprogramming and endothelial dysfunction. Pyruvate kinase (PK), a rate-limiting glycolytic enzyme, catalyzes the conversion of phosphoenolpyruvate to pyruvate, thereby linking to ATP production and subsequent acetyl-CoA formation for entry into the TCA cycle^13^. Among its isoforms, PKM2 is predominantly expressed in proliferating and cancerous cells. It exists either as a tetrameric form that supports glycolytic activity or as a nuclear dimer that regulates gene expression, thereby playing a pivotal role in cancer metabolism and progression^13–16^. PKM2 is upregulated in KSHV-infected blood endothelial cells (BECs) via HIF1α signaling to promote glycolysis and angiogenesis^17^; however, its relevance to KS pathogenesis remains unclear in lymphatic endothelial cells (LECs), the recognized origin of KS. Moreover, recent studies highlight the metabolic diversity required at different stages of KSHV replication and pathogenesis. For instance, glutamine-mediated anaplerosis supports the TCA cycle and is essential for the survival of TIME cells infected with KSHV^18^, whereas viral protein such as vFLIP suppresses aerobic glycolysis through NF-κB signaling, in apparent contradiction to previous findings^19^. Notably, most studies on KSHV-induced metabolism have been conducted under hypoxic, in vitro conditions, often using BECs rather than models that reflect the LECs origin and in vivo context of KS. Together, in the context of the Warburg effect, recent reports highlight the metabolic diversity required at different stages of KSHV replication and pathogenesis^18,19^. Therefore, these observations highlight a critical knowledge gap and underscore the need to investigate the role of PKM2–acetyl-CoA axis in KSHV-induced metabolic plasticity and KS progression.

Here, we elucidated how KSHV governs a high level of intracellular acetyl-CoA by modulating PKM2 in KS. In finer detail, our study has demonstrated that the interaction between KSHV viral interferon regulatory factor 3 (vIRF3) and PKM2 significantly enhances PK activity by facilitating PKM2 tetramerization, leading to elevated intracellular acetyl-CoA level. This increased level of acetyl-CoA is primarily attributed to augmented pyruvate catabolism rather than to FAO pathway or glutaminolysis. Moreover, the human total protein acetylation microarray revealed that increased acetyl-CoA levels promote SMAD5 acetylation, thereby inducing the endothelial-to-mesenchymal transition (EndMT) in KSHV-infected LECs. Notably, the xenograft model of recombinant KSHV-infected LECs caused secondary tumorigenesis, whereas PKM2-binding-defective mutant KSHV failed do so. Importantly, we have discovered a short viral peptide, VDP, that acts as a potent and selective PKM2 antagonist by competing with vIRF3 for PKM2 interaction, resulting in marked tumor regression.

## Materials and methods

### Study design

Sample sizes for all experiments were performed with *n* ≥ 3. In mouse xenograft models, all control and xenografted mice in each experiment were sex and age matched. For peptide treatment to xenograft model, randomization was performed at after tumor inoculation.

### Cell culture and cell line construction

293T, SLK, and iSLK cells were cultured in Dulbecco’s modified Eagle’s medium supplemented with 10% fetal bovine serum (FBS) and 1% penicillin–streptomycin (Corning). Primary LEC, LEC-E6/E7-V5-vector, LEC-E6/E7-V5-vIRF3-WT, and LEC-E6/E7-V5-vIRF3-MT cells were maintained in VascuLife VEGF Endothelial Medium Complete Kit (Lifeline Cell Technology). For LEC-E6/E7 cell lines, 200 μg/ml G418 and 5 μg/ml puromycin were added into the culture medium. Transient transfection was performed using PEI (Sigma), FuGENE HD (Promega), and PEIpro (Polyplus) according to the manufacturers’ protocols.

### Chemicals and antibodies

Chemicals were purchased from the following manufacturers: doxycycline, sodium butyrate, and D-glucose solution from Sigma; carbonyl cyanide 4-(trifluoromethoxy) phenylhydrazone (FCCP), rotenone, antimycin A1, 2-deoxy-D-glucose (2-DG), and 3,5-Dichloro-2-hydroxy-N-(4-methoxy[1,1′-biphenyl]-3-yl)-benzenesulfonamide (BMS-303141) from Cayman Chemical; and oligomycin complex from Biovision. Primary antibodies were purchased from the following manufacturers: anti-acetylated-lysine (6952S) from Cell Signaling Technology; anti-KSHV LANA (LNA-1; 4013) and anti-KSHV LANA (ab267352) from Abcam; anti-GST (sc-138), anti-lamin A/C (sc-625), anti-KSHV ORF45 (sc-53883), anti-KSHV K8.1 (sc-65446), anti-KSHV K8 (sc-57889), anti-SMAD2 (sc-101153), anti-SMAD3 (sc-101154), anti-SMAD5 (sc-101151), anti-vimentin (sc-6260), anti-fibronectin (sc-8422), anti-N-cadherin (sc-59987), anti-α-SMA (sc-53142), and anti-acetyl H3 (sc-32268) from Santa Cruz; anti-β-actin (A-5441), anti-FLAG (F1804), anti-KSHV ORF59 (MABF2752), and anti-α-tubulin (GTU-88) from Sigma; anti-V5 (MA5-15253) and anti-KSHV LANA2 (MA1-16663) from Invitrogen; anti-VE-cadherin (555661) from BD Pharmingen; anti-HA (M180-3) from MBL; anti-PKM1 (15821-1-AP) and anti-PKM2 (15822-1-AP) from Proteintech; anti-SMAD7 (NBP2-24710) and anti-KSHV LANA2 (NB200-167) from Novus Biologicals; and anti-alpha smooth muscle actin (449004) from Synaptic Systems. Anti-LYVE-1 (orb233665) was obtained from Biorbyt. TAT, TAT-VDP, and TAT-mVDP peptides were synthesized by Bionics.

### Plasmid construction

The pCMV-HA-hPKM2 plasmid was kindly provided by Dr Nobuhiro Tanuma (Division of Cancer Chemotherapy, Miyagi Cancer Center Research Institute, Natori, Japan). The vIRF3-MT (Δ250-280) mutant was generated by sequential deletion of ten amino acids from alanine 250 with QuikChange XL Site-Directed Mutagenesis Kit (Agilent). The primer pairs used for vIRF3 deletion mutation are described below; vIRF3 (Δ250-260) forward 5’-GCTTGATAAAGAATCTATGGA CATGCTAATGA-3’, vIRF3 (Δ250-260) reverse 5’-TCATTAGCATGTCCATAGATTCTTTAT CAAGC-3’, vIRF3 (Δ260-270) forward 5’-GCTTGATAAAGAATCTGAAGACCTCTTTGATC -3’, vIRF3 (Δ260-270) reverse 5’-GATCAAAGAGGTCTTCAGATTCTTTATCAAGC-3’, vIRF3 (Δ270-280) forward 5’-GCTTGATAAAGAATCTGAGGATGTTATCGCAA-3’, vIRF3 (Δ270-280) reverse 5’-TTGCGATAACATCCTCAGATTCTTTATCAAGC-3’. GST-tagged vIRF3 250-260, 260-270, 270-280 were subcloned into pEBB-GST vector with *BamHI* and *NotI* restriction site using following primers; vIRF3 (250-260) forward 5’- GATCCGCCACCATGGC ATGTGCATTGATGTACCACGTGGGACAGGAGTAGGC-3’, vIRF3 (250-260) reverse 5’- G GCCGCCTACTCCTGTCCCACGTGGTACATCAATGCACATGCCATGGTGGCG-3’, vIRF3 (260-270) forward 5’- GATCCGCCACCATGGAGATGGACATGCTAATGAGG GCGATGTGCGATTAGGC-3’, vIRF3 (260-270) reverse 5’-GGCCGCCTAATCGCAC ATCGCCCTCATTAGCATGTCCATCTCCATGGTGGCG-3’, vIRF3 (270-280) forward 5’-GATCCGCCACCATGGATGAAGACCTCTTTGATCTGCTTGGCATCCCATAGGC-3’, vIRF3 270-280 reverse 5’-GG CCGCCTATGGGATGCCAAGCAGATCAAAGAGGTC TTCATCCATGGTGGCG-3’. PKM2 truncated mutants were generated in pCMV3 vector with *EcoRI* and *NotI* restriction site using following primers; PKM2 forward 5’-GCGAATTCCAGCCACCATGTCGAAGCCCCATAGTG -3’, PKM2 225 reverse 5’-GCAACTAAGCGGCCGCTTAGTCCTTCTCCGACACAGC-3’, PKM2 402 reverse 5’- GAATCTTAGCGGCCGCTTACGCCAGGCGGCGGAGTTC-3’, PKM2 210 forward 5’-GAGAATTCGCGCCACCATGAACCTTCCTGGGGCTGCT-3’, PKM2 107 forward 5’-GAGAATTCGCGCCACCATGCCCGTTGCTGTGGCTCTA-3’, PKM2 reverse 5’-GATT CTATGCGGCCGCTCACGGCACAGGAACAAC-3’. The pcDNA3.1-PKM2-V5-6xHis plasmid was generated using *EcoRI* and *NotI* restriction site and PCR with following primer pairs; PKM2 forward 5’-GCGAATTCCAGCCACCATGTCGAAGCCCCATAGTG-3’, PKM2 reverse 5’-GATTCTATGCGGCCGCTCACGGCACAGG AACAAC-3’

### Large-scale immunoprecipitation and mass spectrometry analysis

LEC-E6/E7 cells expressing vector or vIRF3 were lysed and subjected to immunoprecipitation (IP) using an anti-V5 antibody, followed by silver staining. Precipitated proteins were separated on a 4–12% gradient gel, fixed overnight, and processed using standard silver staining protocols. Differential bands present in the vIRF3-expressing lane but absent in the vector control were excised and submitted to the Taplin Mass Spectrometry Facility (Harvard Medical School) for liquid chromatography–mass spectrometry (LC–MS) analysis.

### Generation of recombinant KSHV clones

The vIRF3 mutant (Δ250-280) and its revertant were generated by bacterial artificial chromosome-based homologous recombination using the KSHV clone BAC16-vIRF3-3xFLAG in *Escherichia coli* GS1783, as previously described^20^. Recombinant KSHV clones were validated by Sanger DNA sequencing and *NheI* digestion, followed by pulsed-filed gel electrophoresis analysis.

### Generation of shRNA construct and lentivirus

The short hairpin RNA (shRNA) targeting SMAD5 was cloned into lentiviral vector pLKO.1 (*AgeI* and *EcoRI)* and packaged in 293T cells using standard third-generation lentiviral components. Viral supernatants were collected 72 hours post-transfection, filtrated (0.4 μm), concentrated by centrifugation (90,000g, 2 hours), and re-suspended in phosphate-buffered saline (PBS). Cells were transduced in the presence of 0.8 μg/ml of polybrene. The SMAD5-targeting sequences used in this study included shSMAD5#1 5’- GCCGAGTAAATGTGTCACTAT-3’ and shSMAD5#2 5’- GCCTAAACATTGGTGTTCAAT-3’.

### Establishment and characterization of iSLK-BAC16 cell lines

The iSLK cell lines harboring BAC16-Flag-vIRF3-WT, vIRF3-Δ250-280 (vIRF3-MT), or its revertant (REV-vIRF3-MT) were generated as previously described^21^. Briefly, iSLK cells were transfected with each BAC16 construct using FuGENE HD and selected with 1 mg/ml hygromycin B. The GFP positive, hygromycin-resistant clones were established after 3 weeks. Reactivation was induced with 1 μg/ml doxycycline and 1 mM sodium butyrate for 72 hours. Cells were harvested for immunoblotting (IB) with anti-Flag antibody, and supernatants were filtered (0.45 μm). Virion-associated DNA purification was purified by proteinase K digestion and phenol–chloroform extraction, followed by KSHV ORF11-specific real-time quantitative PCR (qPCR) to quantify viral genome copies based on a BAC16 DNA standard curve.

### Subcellular fractionation assay

Cells were lysed in a fractionation buffer (20 mM HEPES [pH 7.4], 10 mM KCl, 2 mM MgCl_2_, 1 mM EDTA, 1 mM EGTA, 1 mM phenylmethylsulfonyl fluoride) and then passed through a 27 G needle (30x). The nuclear pellet was obtained after centrifugation at 720g for 15 min. The supernatant was cleared by centrifugation at 12000g for 15 minutes (cytoplasmic fraction). The nuclear pellet was lysed in NP-40 lysis buffer (150 mM NaCl, 0.5% NP40, 50 mM Tris [pH 8.0]). Both nucleus and cytoplasmic fractions were mixed with the sample buffer and were analyzed by IB.

### IP and GST pull-down

Indicated cells were transfected with noted combination of plasmids or not. Cells were harvested and lysed in NP-40 lysis buffer supplemented with protease inhibitor cocktail tablet (Thermofisher). Cell lysates were immunoprecipitated against indicated proteins and analyzed by IB.

### PKM2 cross-linking and gradient PAGE gel running

293T cells were transfected with indicated combination of plasmids and harvested 48 hours after the transfection. Cells were lysed with NP-40 lysis buffer supplemented with protease inhibitor and treated with 0.15% glutaraldehyde for 4 minutes at room temperature. Gradient SDS–PAGE was performed with NuPAGE 4–12% Bis-Tris gel (Invitrogen) and analyzed by IB. A GST pull-down assay was performed by precipitating GST-tagged proteins with glutathione Sepharose 4B bead (Cytiva), followed by IB with indicated antibodies.

### PK activity assay

The PK activity was measured using the Pyruvate Kinase Assay Kit (Sigma Aldrich) according to manufacturer’s instructions. LEC-E6/E7 cells expressing vector, vIRF3-WT, or vIRF3-MT were lysed in assay buffer, and cleared lysates were incubated with the assay mix in a 96-well plate. Absorbance at 570 nm was measured using a Varioskan LUX microplate reader (Thermo Scientific), and the activity was calculated per the manufacture’s protocol.

### Acetyl-CoA assay

Acetyl-CoA assay was performed using the Acetyl-CoA assay kit (Sigma Aldrich) following the manufacturer's protocol. Briefly, LEC cell lines were harvested and lysed with 50 μl of acetyl-CoA assay buffer. Lysates were deproteinized with perchloric acid and neutralized with KOH. The supernatants were transferred into an opaque 96-well plate and mixed with the reaction mixture; the fluorescence intensity was measured at λ_ex_ = 535 nm and λ_em_ = 587 nm. The acetyl-CoA concentration was calculated using the standard curve of serially diluted acetyl-CoA standard sample.

### Lactate and ATP assay

Lactate and ATP levels were measured using the Lactate-Glo and CellTiter-Glo assay (Promega) according to the manufacturer’s instructions. Cells were seeded in 96-well plates and incubated for 24 hours. Culture media and cells were collected separately for lactate and ATP analysis, respectively. Luminescence was measured using the Varioskan LUX microplate reader, and concentrations were calculated based on standard curves generated from the serial dilutions of lactate or ATP standards.

### Metabolite extraction and LC–MS-based metabolomics analysis

LEC-E6/E7 cell lines respectively bearing vector, vIRF3-WT, and vIRF3-MT were seeded on the 100-mm cell culture dish for metabolic analysis. After 48 hours, cells were harvested with cold 80% methanol and homogenized with 0.1 mm zirconia/silica beads using Bead Ruptor Elite (Omni). The bead and cell debris were discarded by centrifugation at 13,000 rpm for 10 minutes. The supernatant was transferred into a cryotube and lyophilized with liquid nitrogen. The samples were stored at −80 °C before LC–MS analysis. For quantitative comparison, metabolite abundances were normalized to the viable cell number during harvest, which was determined in the same cultures using a hemocytometer (trypan blue exclusion). LC–MS-based metabolomics was used as described in a previous report^22^. Extracted metabolites were separated on a Cogent Diamond Hydride TYPE-C column (gradient 3), and LC–MS analysis of metabolites was performed. The mobile phase consisted of the following: solvent A (ddH_2_O with 0.2% formic acid) and solvent B (acetonitrile with 0.2% formic acid). The gradient used was as follows: 0–2 minutes, 85% sol B; 3–5 minutes, 80% sol B; 6–7 minutes, 75% sol B; 8–9 minutes, 70% sol B; 10–11.1 minutes, 50% sol B; 11.1–14 minutes, 20% sol B; 14.1–24 minutes, 5% sol B; followed by a 10-minutes re-equilibration period at 85% sol B at a flow rate of 0.4 ml/min. An Agilent Accurate Mass 6230 time-of-flight coupled with an Agilent 1290 Infinity II LC system was used. Detected ions were deemed metabolites on the basis of unique accurate mass retention time identifiers for masses exhibiting the expected distribution of accompanying isotopologues. Metabolite identities were searched using a mass tolerance of <  0.005 Da. The abundance of extracted metabolite ion intensities was extracted using MassHunter Profinder B.08.00 and Agilent Qualitative Analysis B.07.00 (Agilent Technologies).

### Isotopologue analysis using U-^13^C glucose

LEC-E6/E7 cell lines bearing vector, vIRF3-WT, and vIRF3-MT were cultured in a conditioned medium supplemented uniformly labeled ^13^C (U-^13^C) glucose (MedChemExpress, HY-B0389A) for 24 hours. Metabolite extraction and detection were conducted as previously described. The extent of isotope labeling was determined by dividing the summed peak height ion intensities of all isotopologues, expressed in percentage. Label-specific ion counts were corrected for naturally occurring ^13^C species (i.e., [M + 1] and [M + 2]). The relative abundance of each isotopologue was determined by diving the peak height ion intensity of each isotopologue form (corrected for naturally occurring ^13^C species as above) by the summed peak height ion intensity of all isotopologues. Fraction enrichment represents the percentage of each isotopologue of metabolites.

### Seahorse XFp aessay

Extracellular acidification rate (ECAR) and oxygen consumption rate (OCR) were measured using Seahorse XFp glycolysis and Mito Stress test Kits (Agilent) on the XF HS Mini Analyzer, following the manufacturer’s protocols. LEC-E6/E7-V5-vector, LEC-E6/E7-V5-vIRF3-WT, and LEC-E6/E7-V5-vIRF3-MT were seeded in XF Seahorse mini plate and incubated overnight. For glycolysis stress assay, cells were incubated for an hour at 37 °C in Seahorse XF DMEM medium (Agilent) without supplementation of glucose and CO_2_. Cells were then analyzed according to the manufacturer's protocol with the following concentration of each chemical: glucose (10 mM), oligomycin (5 μM), and 2-DG (100 mM). For the Mito Stress test, cell culture medium was replaced with XF DMEM medium supplemented with 10 mM glucose, 10 mM L-glutamine, and 1 mM sodium pyruvate and incubated for an hour at 37 °C without CO_2_. Cells were then analyzed according to the manufacturer's protocol with following concentration of each chemical: oligomycin (5 μM), FCCP (1 μM), rotenone/antimycin A1 (3 μM), and 2-DG (100 mM).

### Protein acetylation array

Protein acetylation profiling was performed using the Human Protein Acetylation Array G8000 (RayBiotech, AAH-ACK-G8000) following the manufacturer’s protocol. Lysates from each cell line were applied to the array, followed by detection with Cy3-conjugated streptavidin. The processed arrays were scanned by RayBiotech. Raw data were processed in R (version 4.4.1) using custom scripts. After normalization to total protein levels, intensity values were filtered and converted into integer counts for DESeq2 analysis. Proteins with a log2 fold change (log2FC) > 1.5 and adjusted *P*-value ≤ 0.05 were considered significantly upregulated, whereas those with log2FC < −1.5 and *P*-value ≤ 0.01 were classified as significantly downregulated.

### Immunofluorescence assay

Cells grown on a glass disc were fixed with 4% paraformaldehyde, permeabilized with 0.1% Triton X-100, and indicated with primary antibodies, followed by FITC-, rhodamine-, or Alexa Fluor 568-conjugated secondary antibodies. Slides were mounted with DAPI-containing medium and imaged using an LSM-700 confocal microscope (Carl Zeiss).

### 3D spheroid invasion assay

LEC spheroids were generated in ultra-low attachment, U-bottom, 96-well plates and embedded in a 3 mg/ml Matrigel matrix. After 3 days of culture, spheroids were fixed with acetone/methanol (1:1), blocked, and stained with indicated antibodies. Z-stack images were acquired using the LSM-700 confocal microscope. Spheroid invasion was quantified using ImageJ the software according to the previous report^23^. Briefly, images were converted to 8-bit grayscale and cropped to define the region of interest. To quantify the extent of invasion, images were segmented into three distinct regions, core (masked as green), intermediate (masked as blue), and edge (masked as red), based on appropriate pixel intensity thresholds. The area of each segmented mask was measured, and the masks were subsequently merged into composite images for visual verification.

### Transwell cell migration assay

LEC cells expressing vector, vIRF3-WT, or vIRF3-MT were seeded in a 24-well plate and incubated with or without 3 μM SIS3-HCl. Transwell migration assays were performed using 8-μm-pore inserts with serum-free medium in the upper chamber and 10% FBS in the lower chamber. After 16 hours, migrated cells were fixed, stained with crystal violet, and imaged using the solution containing 1% formaldehyde and 25% methanol. The non-migrated cells on the top side of transwells were removed with cotton swab and washed with 1X PBS. The migrated cells on the bottom side of transwells were counted and imaged with EVOS-M5000 imaging system.

### Chorioallantoic membrane invasion assay

Chorioallantoic membrane (CAM) invasion assays were performed using chick embryo under 14 days of development; ethical approval was not required due to the absence of nociception. Fertilized eggs were incubated at 37.5 °C with 70% humidified atmosphere in Rcom D-50 incubator (Autoelex). On embryonic day 10, cells were seeded onto the CAM within silicone rings, and 0.2 mg of TAT or TAT-VDP peptides were applied at 24 and 48 hours post-seeding. On embryonic day 14, CAM tissues were harvested, fixed in 4% paraformaldehyde, and processed for H&E staining and immunohistochemistry (Genoss).

### Protein complex structure prediction

AlphaFold2-multimer version 3.0 in ColabFold was used for the prediction of the VDP–PKM2 complex structure with three recycles and five structures predicted^24^. The interface energy was calculated using Rosetta energy functions to identify the best model^25^.

### Molecular dynamics simulations

All-atom molecular dynamics simulations were performed using the GROMACS 2021 package^26^. The simulations of the fast relaxed complex (VDP–mPKM2) with the lowest interface energy (model 3) were performed using the AMBER99SB-ILDN force field for protein and TIP3P for water^27^. LINCS constraints were applied to all bonds involving hydrogen atoms. The long-range electrostatic interactions were computed using the Particle Mesh Ewald method with a Fourier grid spacing of 0.16, and a cutoff distance of 12 Å was considered for the van der Waals interactions. A suitable number of Cl^−^ counterions were added to the system to neutralize the system. Constant pressure and temperature (1 atm and 300 K, respectively) were maintained in the system, with the non-bonded pair list updated every 10 fs and an integration time step of 100 ps. Upon the addition of ligands, the system was equilibrated by pressure and temperature coupling for 100 ps. Finally, a 50 ns molecular dynamics simulation was performed. The root mean square deviation was evaluated using the appropriate GROMACS tools. The binding energy of the ligand was estimated by the single trajectory protocol with the Molecular Mechanics–Poisson Boltzmann Surface Area method with the gmx_MMPBSA module^28^. The last 10 ns of the 50 ns simulation, at an interval of 1000 ps, was used for the calculation of enthalpy of binding of the ligand to protein. All the structures were visualized using Chimera^29^.

### PKM2 protein purification

Expi293F cells were transfected with the pcDNA3.1-PKM2-V5-6xHis plasmid using ExpiFectamine 293 reagent following the manufacturer’s manual. Expi293F cells were cultured at 37 °C, 8% CO_2_ atmosphere. After 3 days of transfection, cells were lysed for Ni-NTA protein purification. The lysates were applied to column filled with Ni-NTA superflow resin (Qiagen) and incubated for an hour at 4 °C. The resin was washed, and bound proteins were eluted according to manufacturer’s guidelines. The isolated His-tagged PKM2 was further loaded onto a Superdex 75 Increase 10/300 GL column and eluted in a buffer containing 25 mM Tris-HCl [pH 7.4], 100 mM NaCl.

### Biolayer interferometry

The binding of PKM2 to TAT-VDP/mVDP was measured by biolayer interferometry (BLI) experiments using the BLItz system (ForteBio). Purified PKM2 was immobilized onto the Ni-NTA biosensor chip and washed using the kinetic buffer (25 mM Tris-HCl [pH 7.4], 100 mM NaCl). Furthermore, various concentrations of the peptide were reacted with the sensor and washed using the kinetic buffer. The sensograms were analyzed by the ‘global fitting’ method in the BLItz Pro 1.2 software (ForteBio) to calculate the *K*_*D*_ (equilibrium dissociation constant), *k*_*a*_ (association rate constant), and *k*_*d*_ (dissociation rate constant) values. The raw sensograms were plotted in Prism 5.

### Immunohistochemistry

After killing the mice, organs and tumor were dissected and immediately fixed with 4% paraformaldehyde. After 24 hours of fixation, tissue and tumor samples were further processed for immunohistochemistry or fluorescence scanning by Genoss.

### Xenograft mouse model

Recombinant KSHV-infected LEC-E6/E7 cells at passage 7–9 were used for xenograft experiments, with no biologically meaningful passage-dependent changes observed within the early-passage range. Recombinant KSHV-infected LECs were washed with PBS and injected subcutaneously into 7-week-old female athymic NCr-nu/nu mouse (Koatech). Each group used five mice. TAT or TAT-VDP peptides were subcutaneously administered to the cell injection site at a dose of 0.2 mg per injection, twice per week after 3 days from cell inoculation. Inhibition of tumor growth was monitored daily for 30 days by comparing body weight and tumor volume between TAT-treated and TAT-VDP-treated mice. The volume of tumor was calculated using the following equation: tumor volume = 1/2 (length × width^2^)^30^. After 30 days of monitoring, TAT-VDP was administered to TAT-treated group to evaluate tumor regression. All data were analyzed by GraphPad Prism software and one-way analysis of variance (ANOVA) test.

## Results

### KSHV vIRF3 interacts with PKM2 in LECs

**Fig. 1:**
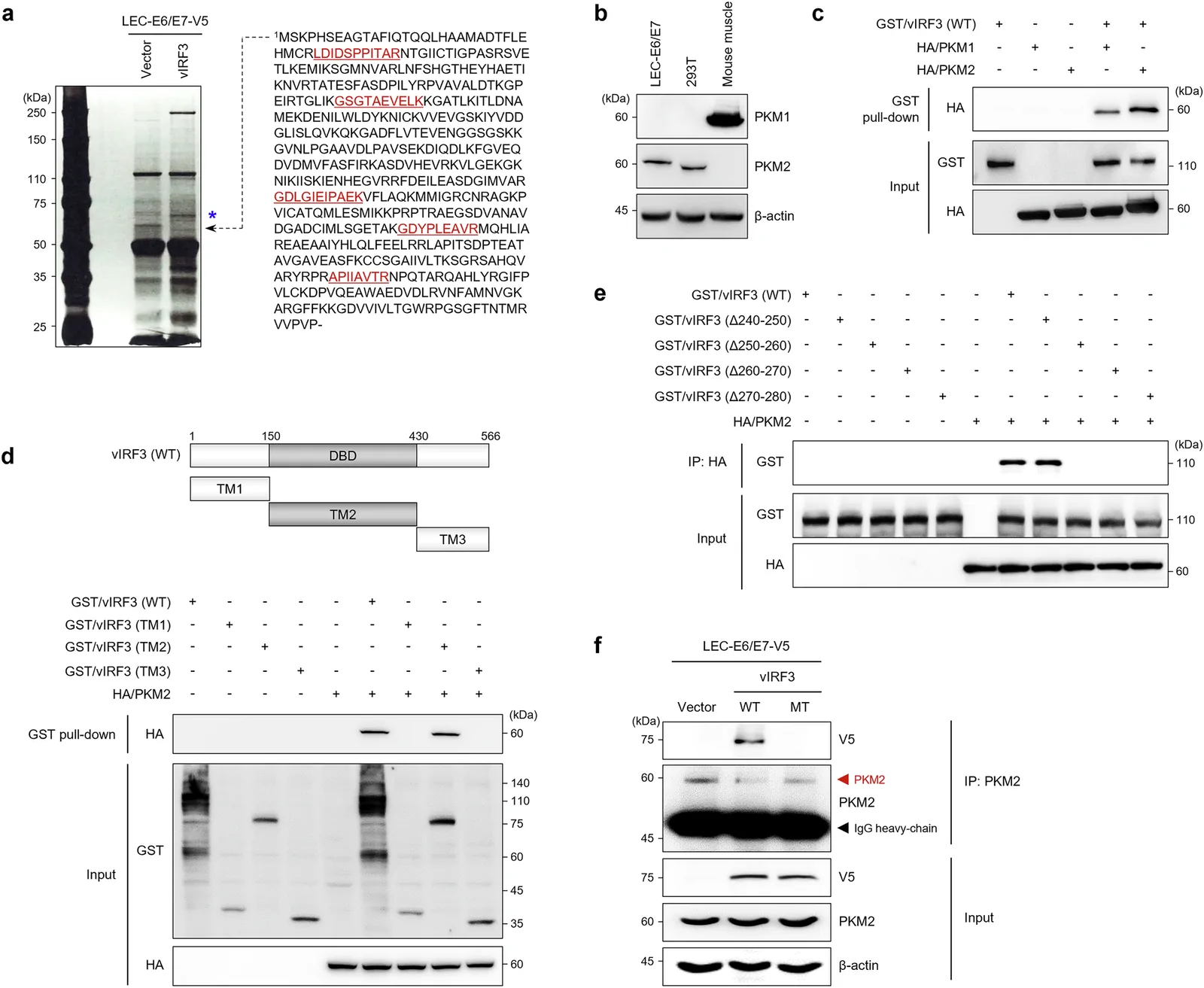
KSHV vIRF3 interacts with endogenous PKM2. **a** PKM peptide sequences identified by mass spectrometry are highlighted in red. The vIRF3 band is denoted by a blue asterisk. **b** Lysates from LEC, 293T, and mouse muscle tissue were IB with isoform-specific antibodies against PKM1 or PKM2, with β-actin serving as an internal loading control. **c** 293T cells were transfected with vIRF3-WT along with PKM1 or PKM2. Subsequently, cell lysates were subjected to a GST pull-down assay, followed by IB with an anti-HA antibody. **d** After transfection of 293T cells with the indicated plasmids combination, cell lysates were used for GST pull-down assay, followed by IB with an anti-HA antibody. **e** The indicated vIRF3 deletion mutants were co-transfected with PKM2 and subjected to IP using anti-HA antibody. **f** Cell lysates from LEC expressing vector, vIRF3-WT, and vIRF3-MT were subjected to IP using anti-PKM2 antibody, followed by IB with anti-V5 antibody.

Our previous study reported that KSHV vIRF3 is expressed in various lesions of KS tissue and induces KS-like spindle morphology as well as lymphangiogenesis^21^. To identify the vIRF3-interacting partners in LECs, we performed a mass spectrometry analysis using V5-tagged vIRF3-expressing LECs. A polypeptide with a molecular mass of ~60 kDa, corresponding to pyruvate kinase M (PKM), was found as a potential novel binding partner of vIRF3 (Fig. 1a). Although both PKM1 and PKM2, the isoforms of PKM, catalyze the final step of glycolysis, their preferred expression in cell types differs. PKM1 is found in terminally differentiated tissues such as the muscle and brain, whereas PKM2 predominates in highly proliferative cells, such as stem cells, embryonic cells, and tumor cells^15,16^. Interestingly, a GST pull-down assay revealed that although vIRF3 interacts with both PKM1 and PKM2, only PKM2 is expressed in LECs (Fig. 1b, c). Therefore, we focused on the vIRF3–PKM2 axis in LECs. Detailed binding assays revealed that the vIRF3_250-280_ region was specifically responsible for its interaction with PKM2 (Fig. 1d, e and Supplementary Fig. 1a). Based on these findings, we generate a vIRF3 mutant (Δ250-280, referred to as vIRF3-MT) that no longer interacts with PKM2, serving as a negative control. To verify the interaction of endogenous PKM2 with vIRF3, we generated LEC lines which are ectopically expressing V5-tagged vIRF3-WT (wild-type) or vIRF3-MT, respectively. The IP experiments showed that vIRF3-WT interacts with endogenous PKM2, but vIRF3-MT does not (Fig. 1f). Given that vIRF3 is known to bind to three different cellular proteins, namely IRF7, HIF1α, and HDAC5^21,31,32^, we examined whether vIRF3-MT loses known binding partners of vIRF3. vIRF3-MT still interacts with IRF7, HIF1α, and HDAC5 but does not interact with PKM2 (Supplementary Fig. 1b). These findings suggest that vIRF3-MT is genetically and functionally separated from interferon or other related signaling pathways, making it an effective negative control for studying the effects of vIRF3–PKM2 interaction.

### KSHV vIRF3–PKM2 interaction facilitates tetramerization of PKM2, leading to its enhanced PK activity

**Fig. 2:**
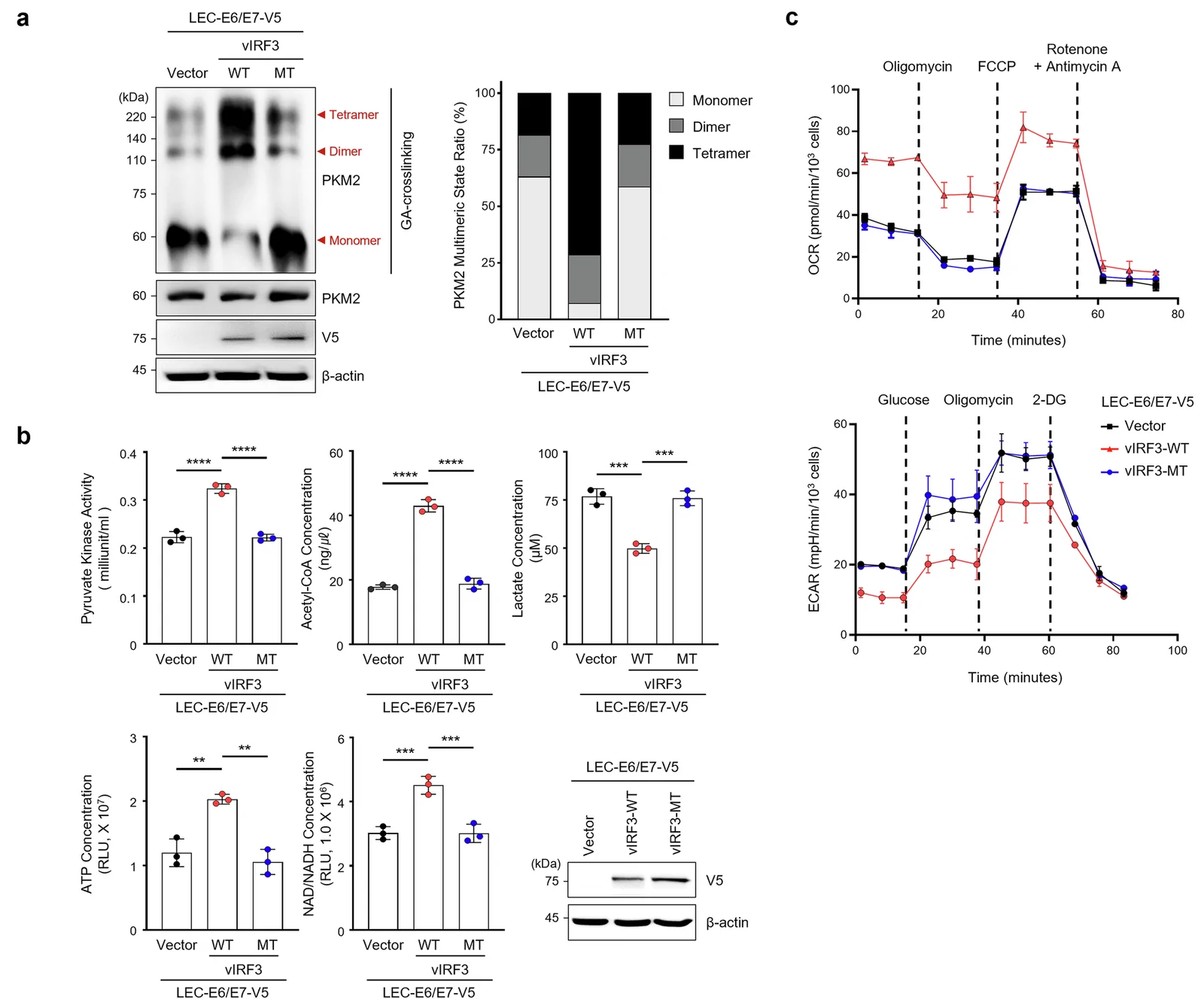
KSHV vIRF3–PKM2 interaction enhances PK activity. **a** Cell lysate from LEC-vector, LEC-V5-vIRF3-WT, and LEC-V5-vIRF3-MT were cross-linked with 0.15% glutaraldehyde. IB with indicated antibodies was performed (left). The graph displays the relative band intensities corresponding to each allosteric state of PKM2 (right). **b** Metabolic profiling was performed in LEC-E6/E7-V5-vector, LEC-E6/E7-V5-vIRF3-WT, and LEC-E6/E7-V5-vIRF3-MT cells. Lysates from the same samples were analyzed for vIRF3 expression with anti-V5 antibody. Data are presented as mean ± s.d. of *n* = 3 independent biological experiments. Statistical significance was assessed using one-way ANOVA; *P* < 0.05 was considered significant. **P < 0.005, ***P < 0.0005, ****P < 0.0001. **c** Cellular oxidative phosphorylation and glycolytic activity were assessed using the Seahorse XFp analyzer. OCR was measured with a Mito Stress Test (upper panel), and ECAR was measured with a Glycolysis Stress Test (lower panel) in LEC-E6/E7 cells expressing vector, vIRF3-WT, or vIRF3-MT. Data were normalized to cell number.

The biological properties of PKM2 oligomers in dimeric and tetrameric forms are markedly different. The tetrameric form of PKM2 mainly acts as PK and promotes the oxidative phosphorylation, whereas the dimeric form of PKM2 serves as a transcriptional co-factor in the nucleus and supports aerobic glycolysis^15,16^. To understand the biological consequences of vIRF3–PKM2 interaction in LECs, we first investigated whether the vIRF3–PKM2 interaction influences the allosteric state of PKM2. When vIRF3 was expressed in LECs, we found that vIRF3-WT greatly enhanced the tetramerization of PKM2, whereas vIRF3-MT did not (Fig. 2a). To further explore the role of vIRF3-mediated PKM2 tetramerization, we measured the PK enzymatic activity. The presence of KSHV vIRF3-WT, but not vIRF3-MT, increased PK activity and acetyl-CoA levels, but reduced lactate concentration (Fig. 2b). In addition, vIRF3-WT enhanced OCRs, with basal respiration, ATP production, proton leak, maximal respiration, and spare respiratory capacity all significantly elevated in LEC-V5-vIRF3 compared with the control groups (Fig. 2c upper). The ECAR value generally shows the changes in the glycolytic rate. The ECAR value was significantly reduced in LEC-V5-vIRF3 (Fig. 2c lower). Taken together, our data indicate that the vIRF3–PKM2 interaction promotes PKM2 enzymatic activity by enhancing its tetramerization, which can alter mitochondrial metabolism.

### KSHV vIRF3–PKM2 interaction significantly elevates the abundance of all of TCA cycle metabolites

**Fig. 3:**
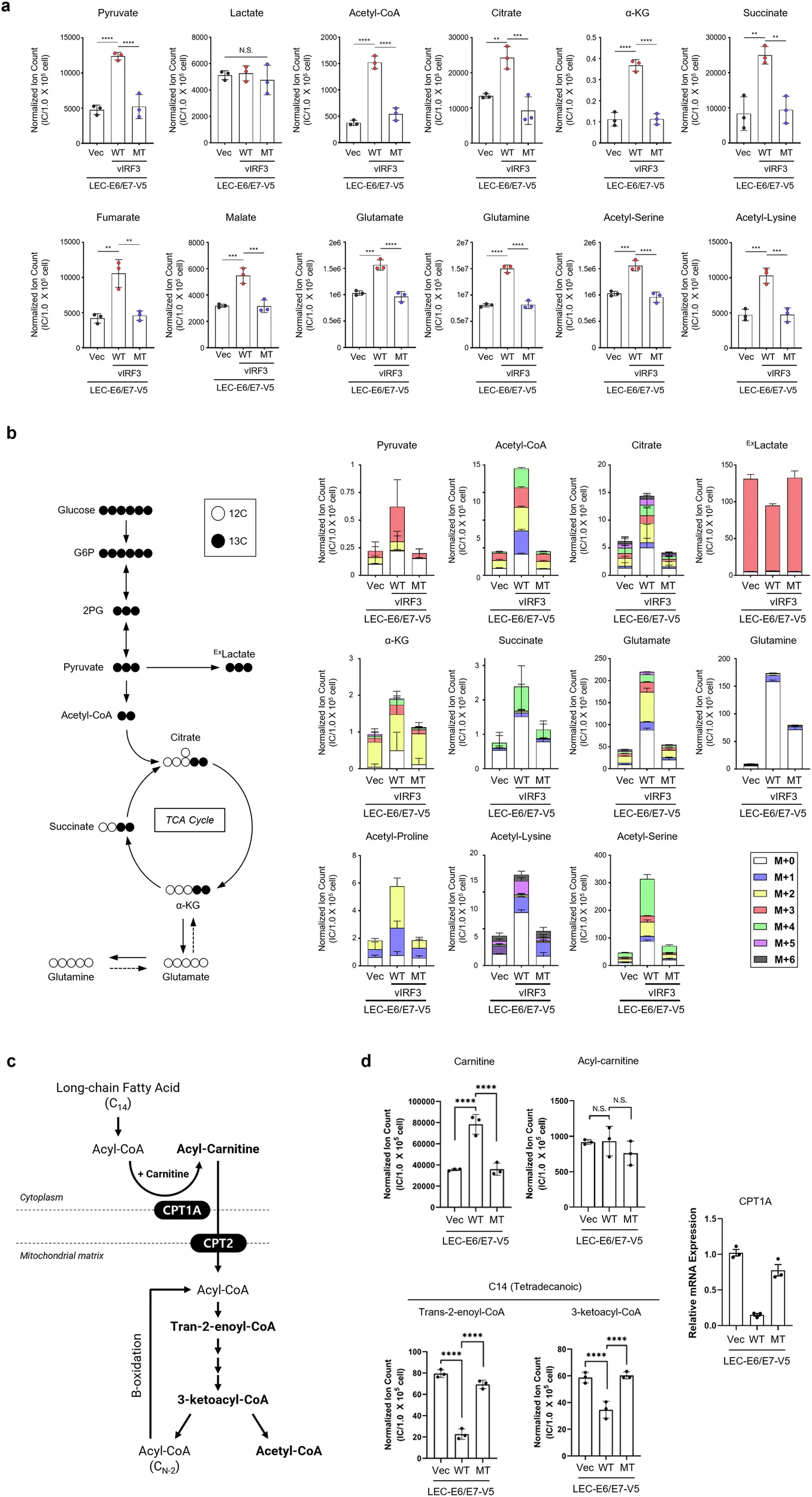
vIRF3–PKM2 interaction significantly increased all TCA cycle metabolites. **a** LEC-E6/E7-V5-vector, LEC-E6/E7-V5-vIRF3-WT, and LEC-E6/E7-V5-vIRF3-MT were harvested and lysed for metabolite extraction. Metabolites from each cell were analyzed by LC–MS. Data are presented as mean ± s.d. of *n* = 3 independent biological experiments. Statistical significance was assessed using one-way ANOVA; *P* < 0.05 was considered significant. N.S. non-significant; *P* > 0.05, **P < 0.005, ***P < 0.0005, ****P < 0.0001. **b** LEC-vector, LEC-V5-vIRF3-WT, and LEC-V5-vIRF3-MT were labeled with ^13^C_6_-glucose in a conditioned medium without other carbon sources. After harvesting, metabolites were extracted and analyzed using LC–MS to measure ^13^C incorporation. **c** Schematic representing long-chain fatty acid oxidation process. **d** Long-chain FAO-related metabolites were analyzed by LC–MS in LEC-E6/E7 cells expressing vector, vIRF3-WT, or vIRF3-MT (left). The mRNA expression of CPT1A was measured by RT-qPCR in LEC-E6/E7-V5-vector, LEC-E6/E7-V5-vIRF3-WT, and LEC-E6/E7-V5-vIRF3-MT (right). Data are presented as mean ± s.d. of *n* = 3 independent biological experiments. Statistical significance was assessed using one-way ANOVA; *P* < 0.05 was considered significant. **P < 0.005, ***P < 0.0005, ****P < 0.0001.

To assess the impact of vIRF3 expression on LEC cellular metabolism, LEC-vector, LEC-V5-vIRF3-WT, and LEC-V5-vIRF3-MT were cultured, and the vIRF3-induced LEC metabolome was analyzed using LC–MS metabolomics with focus on TCA cycle intermediates^22^. Notably, LEC-V5-vIRF3-WT exhibited significantly increased pyruvate and acetyl-CoA levels, but not lactate, compared with LEC-V5-vector or LEC-V5-vIRF3-MT. The enhanced acetyl-CoA level was preferentially utilized for the biosynthesis of TCA cycle intermediates, such as citrate, α-ketoglutarate (α-KG), succinate, fumarate, and malate (Fig. 3a). To further clarify whether TCA cycle intermediates originate from either glycolytic intermediates or glutaminolysis, we performed the isotope tracing experiment using [U-^13^C] glucose as a carbon source. Our results showed that the increased pyruvate levels in LEC-V5-vIRF3-WT mainly displayed M + 3 isotopologue, which is functionally associated with PKM2 activity in converting phosphoenolpyruvate to pyruvate. Consequently, the decarboxylation of M + 2 and M + 3 isotopologue of pyruvate mainly produced M + 1 or M + 2 isotopologue of acetyl-CoA, serving as a substrate for the biosynthesis of the oxidative branch of TCA cycle. This supports the hypothesis that vIRF3–PKM2 interaction activates glycolysis-mediated acetyl-CoA synthesis via increased PKM2 activity and functions as a major route for the TCA cycle intermediate biosynthesis. Separately, we noticed that accumulated glutamate comprised M + 0 and M + 2 isotopologues, which were similar to α-KG, but not to glutamine. This finding inferred that glutamate production is mediated by using α-KG as a substrate (Fig. 3b). Moreover, the elevated level of acetyl-serine in LEC-V5-vIRF3-WT was composed predominantly of the M + 5 isotopologue, which is presumably generated by the condensation of M + 3 serine and M + 2 acetyl-CoA (Fig. 3b). This finding suggests that vIRF3-mediated acetyl-CoA accumulation also serves as a substrate for the acetylation of specific amino acids.

Notably, cytosolic acetyl-CoA can also be supplied by mitochondrial FAO, promoting us to investigate whether vIRF3 reshapes FAO-derived acetyl-CoA production. Carnitine and carnitine palmitoyltransferase 1A (CPT1A) facilitate the mitochondrial import of long-chain fatty acids, which subsequently undergo β-oxidation to generate acetyl-CoA (Fig. 3c). In vIRF3-WT-expressing LECs, total carnitine levels were markedly accumulated, whereas CPT1A expression and the FAO intermediates trans-2-enoyl-CoA and ketoacyl-CoA were markedly reduced. These alterations were not observed in vector or vIRF3-MT-expressing cells (Fig. 3d). This metabolic profile suggests that vIRF3 suppresses CPT1A expression, thereby limiting the mitochondrial import of fatty acylcarnitines. Consequently, carnitine accumulates while FAO flux is attenuated, leading to depletion of downstream FAO intermediates. Consistent with the [U-^13^C] glucose tracing results, these findings indicate that FAO contributes minimally to the expanded acetyl-CoA pool in vIRF3-expressing LECs. Instead, they support a model in which the vIRF3–PKM2 axis predominantly derives acetyl-CoA production through enhanced pyruvate catabolism rather than through FAO.

### KSHV vIRF3–PKM2 enhances intracellular acetyl-CoA production from pyruvate catabolism, leading to SMAD5 acetylation that triggers EndMT

**Fig. 4:**
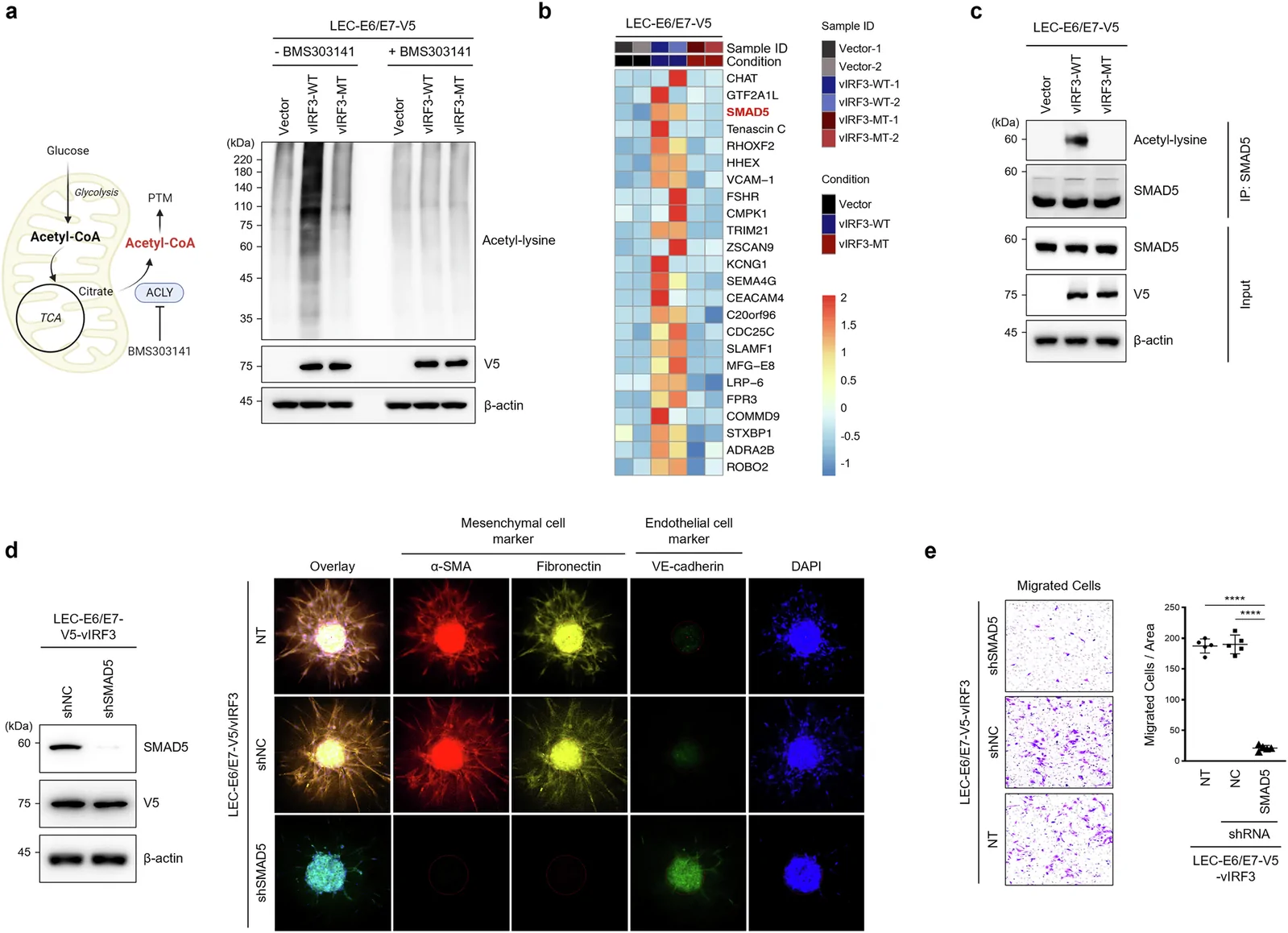
vIRF3–PKM2 increases the total amount of cellular acetyl-CoA in a citrate-dependent manner, thereby enhancing SMAD5 acetylation to promote cell invasion and migration. **a** LEC-E6/E7-V5-vector, LEC-E6/E7-V5-vIRF3-WT, and LEC-E6/E7-V5-vIRF3-MT were treated with the ACLY inhibitor BMS-303141 and analyzed by IB using anti-acetyl-lysine and anti-V5 antibodies. **b** Total protein acetylation was assessed using human protein acetylation microarray. Data were normalized to internal controls and total protein levels. The heatmap shows proteins whose acetylation was significantly upregulated in vIRF3-WT versus vector but downregulated in vIRF3-WT versus vIRF3-MT (log₂FC < −1.5, *P* < 0.01). Rows represent acetylated proteins, columns indicate experimental conditions with replicates, and color scale reflects relative acetylation (blue: low, red: high). **c** LEC-E6/E7 cells expressing vector, vIRF3-WT, or vIRF3-MT were IP with anti-SMAD5 antibody and IB with an acetyl-lysine antibody to assess SMAD5 acetylation. **d** LECs expressing vIRF3-WT were transduced with the lentivirus encoding either control shRNA (shNC) or SMAD5-targeting shRNA (shSMAD5). Cells were harvested 24 hours after transduction and were used for spheroid formation. Spheroids were embedded in a 3D Matrigel matrix for invasion assay and stained with the indicated antibodies. **e** Boyden chamber migration assay was performed in LEC-E6/E7-V5-vIRF3 cells transduced with the indicated shRNAs. Migrated cells were quantified from five randomly selected microscopic fields in each experiment, and data represent three independent experiments. Data are presented as mean ± s.d., *n *= 3 independent biological experiments. Statistical significance was assessed using one-way ANOVA; *P* < 0.05 was considered significant. ****P < 0.0001.

Acetyl-CoA is a crucial metabolite that provides acetyl groups for acetylation reaction of proteins including histones^4^. Especially, cytosolic acetyl-CoA, which mainly participates in lysine acetylation of protein, regulating their activity, stability, or localization, is primarily generated by the translocation of citrate from the mitochondria to the cytoplasm via ACLY^4^. Therefore, three LEC stable cell lines encoding vector, V5-vIRF3-WT, and V5-vIRF3-MT were treated with the ACLY inhibitor BMS-303141, followed by IB with an anti-acetyl-lysine antibody. Consistent with the metabolomics analysis, vIRF3 expression greatly increased the global level of acetylation in LECs, unlike vIRF3-MT or vector. However, treatment with an ACLY inhibitor did not enhance total acetylation levels in LEC-vIRF3-WT, suggesting that the increased acetylation primarily originates from pyruvate catabolism-derived citrate (Fig. 4a).

To examine the impact of vIRF3–PKM2 interaction-mediated acetyl-CoA elevation in LECs, we performed a human protein acetylation array using LEC-E6/E7 cell lines expressing vector, vIRF3-WT, and vIRF3-MT. After analyzing the data, we identified 24 proteins and visualized in a heatmap. Among them, SMAD5, a crucial transcriptional effector of TGFβ–BMP signaling, was selected for further investigation (Fig. 4b). Growing evidence indicates that TGFβ–BMP signaling is a key driver of EndMT-related disease^4,33,34^. For example, a recent study reported that alteration in intracellular acetyl-CoA levels and SMAD7 signaling in BECs are critical for EndMT^35^. Furthermore, LECs expressing vIRF3-WT displayed a spindle-shaped, mesenchymal-like morphology, unlike LECs expressing vector or vIRF3-MT, indicating a loss of adhesion and acquisition of mesenchymal characteristics (Supplementary Fig. 2a). To investigate the effect of vIRF3–PKM2 interaction-mediated elevated acetyl-CoA levels on TGFβ/BMP signaling, we first performed IP using SMAD5 antibody, followed by IB with an acetyl-lysine antibody. We observed that vIRF3-WT expression significantly increased SMAD5 acetylation, but not in vector- or vIRF3-MT-expressing LECs (Fig. 4c). This result suggests that vIRF3–PKM2 interaction enhances SMAD5 acetylation, potentially increasing SMAD5-mediated transcriptional activity and promoting the expression of mesenchymal-associated genes^36–40^. We thus investigated the impact of vIRF3 on typical mesenchymal and endothelial markers. Our results show that vIRF3-WT expression significantly downregulates endothelial markers (LYVE-1 and VE-cadherin) while simultaneously upregulating mesenchymal markers, including vimentin, fibronectin, N-cadherin, and α-smooth muscle actin (α-SMA) (Supplementary Fig. 2b). Conversely, vIRF3-MT had no effect on endothelial markers expression (Supplementary Fig. 2b). Because mesenchymal cells are highly invasive and migratory^39^, we conducted Boyden’s chamber and 3D spheroid invasion assays by knocking down SMAD5 in LEC-E6/E7-V5-vIRF3-WT cells using shRNA specifically targeting SMAD5 (Fig. 4d and Supplementary Fig. 2c). The 3D spheroid invasion assay showed that vIRF3 significantly promoted invasion while inducing mesenchymal markers (α-SMA, fibronectin) and suppressing the endothelial marker VE-cadherin in a SMAD5-dependent manner (Fig. 4d and Supplementary Fig. 2d). Consistent with this, vIRF3-mediated migration absolutely relies on SMAD5 (Fig. 4e and Supplementary Fig. 2e). Importantly, co-IP analysis identified p300 as the major lysine acetyltransferase (KAT) associated with SMAD5 in vIRF3-expressing LECs, whereas no detectable interaction was observed with other well-known KATs—PCAF or GCN5 (Supplementary Fig. 3a). Moreover, pharmacological inhibition of p300 using A-485 markedly reduced SMAD5 acetylation, supporting a model in which vIRF3-induced acetyl-CoA accumulation promotes p300-dependent SMAD5 acetylation (Supplementary Fig. 3b). Collectively, these results indicate that vIRF3–PKM2 interaction induces SMAD5 acetylation, driving mesenchymal reprogramming in LECs.

### The KSHV life cycle is not affected by the vIRF3–PKM2-mediated increase in PK activity

**Fig. 5:**
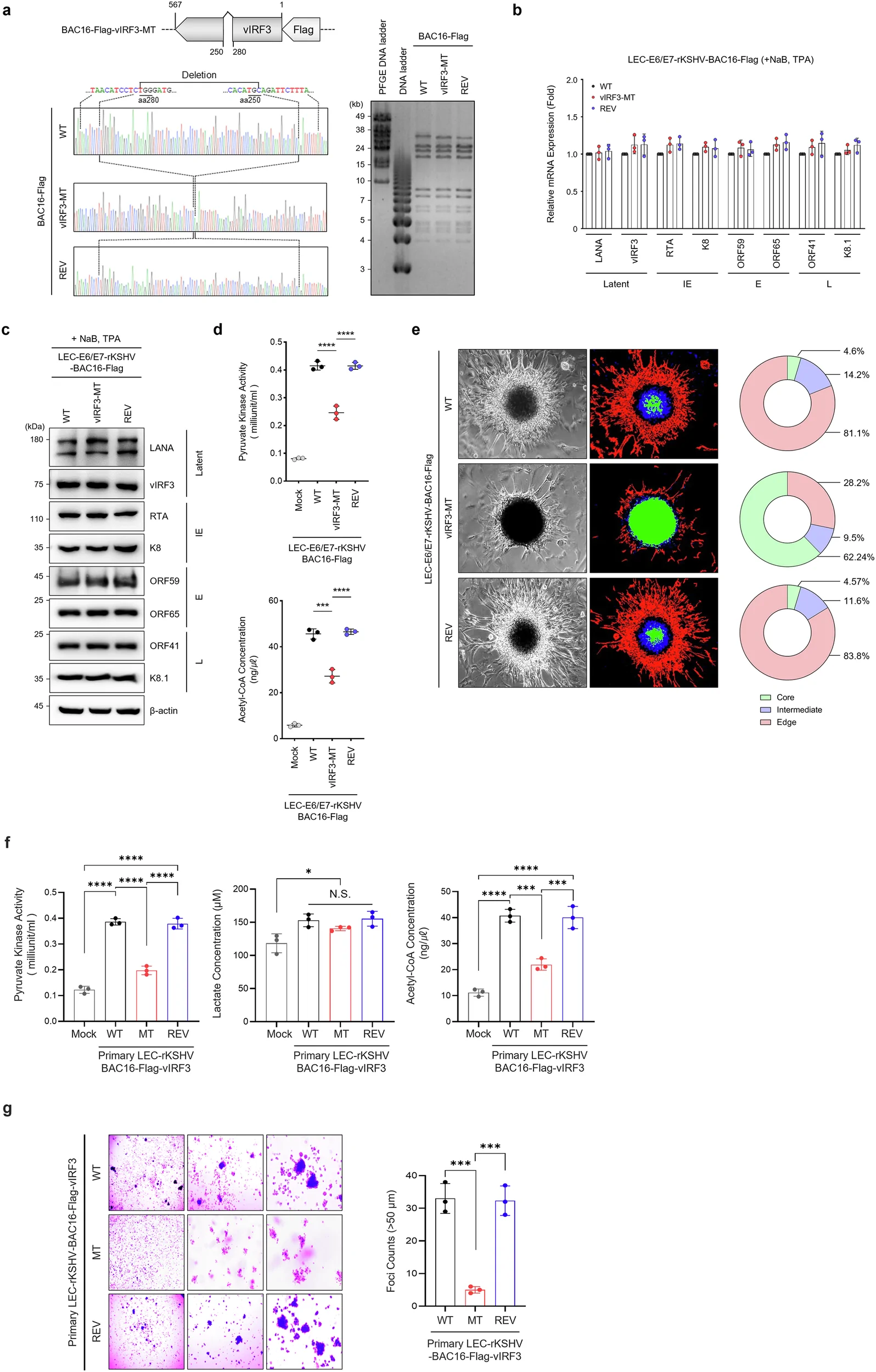
vIRF3-MT, which lacks PKM2 interaction, did not affect virus production and infectivity. **a** Schematic representation of BAC16-Flag-vIRF3 (Δ250-280) construct (upper) and sequencing result of BAC16-Flag-vIRF3 wild-type (WT), vIRF3 (Δ250-280) mutant (MT) and revertant (REV) (lower left). BAC16-Flag-vIRF3-WT, BAC16-Flag-vIRF3-MT, and BAC16-Flag-REV-vIRF3-MT were digested with *NheI* restriction enzyme and analyzed by pulse-field gel electrophoresis (lower right). **b** LEC-E6/E7 cells were infected with recombinant WT, vIRF3-MT, or REV-vIRF3-MT viruses, followed by lytic reactivation using NaB and doxycycline. The mRNA expression of indicated viral genes was quantified by RT-qPCR using viral gene-specific primers. Data are presented as mean ± s.d. of *n* = 3 independent biological experiments. Statistical significance was assessed using one-way ANOVA with a significance threshold of *P* < 0.05; no statistically significant differences were observed among the groups. **c** The expressions of indicated viral proteins were assessed by IB with indicated antibodies. **d** Lysates from LECs infected with the indicated rKSHV were analyzed via PK activity assay and acetyl-CoA assay at 72 hpi. Data are presented as mean ± s.d., *n* = 3 independent biological experiments. Statistical significance was assessed using one-way ANOVA; *P* < 0.05 was considered significant. ***P < 0.0005 and ****P < 0.0001. **e** LEC-E6/E7 cells infected with indicated rKSHV were cultured in ULA plate for spheroid formation and transferred into Matrigel 3D matrix at 72 hpi. Spheroids were allowed to invade into the matrix for 72 hours and representative images were captured. All images were segmented as core (green), intermediate (blue), and edge (red) based on the intensity. The mean area of each segmented region was quantified, and the ratios were calculated using ImageJ software. **f** Primary LECs were infected with recombinant WT, vIRF3-MT, or REV-vIRF3-MT KSHV, followed by PK activity assay, lactate assay, and acetyl-CoA assay at 72 hpi. Data are presented as mean ± s.d. of *n* = 3 independent biological experiments. Statistical significance was assessed using one-way ANOVA; *P* < 0.05 was considered significant. N.S. non-significant. *P* > 0.05, *P < 0.05, ***P < 0.0005, ****P < 0.0001. **g** Soft agar colony formation assay was performed with primary LECs infected with recombinant WT, vIRF3-MT, or REV-vIRF3-MT KSHV at 72 hpi. Colony numbers were quantified from five randomly selected representative fields per sample, and data represent the mean of one sample from three independent experiments. Data are presented as mean ± s.d.; statistical significance was assessed using one-way ANOVA; *P* < 0.05 was considered significant; ***P < 0.0005.

To examine the effect of vIRF3–PKM2 interaction in the KSHV life cycle, we constructed an N-terminally Flag-tagged vIRF3-MT mutant KSHV by deleting amino acids 250-280 in vIRF3 within KSHV BAC16 (BAC16-Flag-vIRF3-MT) using 'scarless' mutagenesis^41,42^. To exclude the possibility of second-site mutations, we also generated a revertant clone restoring the WT vIRF3 sequence (BAC16-Flag-REV-vIRF3-MT) (Fig. 5a). After validation by *NheI* digestion and DNA sequencing (Fig. 5a), we produced infectious recombinant virus using iSLK cell lines carrying Flag-WT, Flag-vIRF3-MT, and Flag-REV-vIRF3-MT KSHV BAC16 clones (Supplementary Fig. 4a). To determine the effect of vIRF3-MT mutant on the viral gene expression and infectious virus production, we induced KSHV lytic reactivation and measured both virus production and the expression of the latent (LANA, vIRF3), immediate–early (RTA, K8), early (ORF59, ORF65), and late (ORF41, K8.1) viral proteins. We found that vIRF3-MT mutant KSHV produces comparable amounts of virus compared to WT KSHV, suggesting that vIRF3-MT mutant does not affect viral replication or gene expression (Supplementary Fig. 4b–d). Consistently, infection of LEC-E6/E7 with equal amounts of these three viruses shows no difference in infectivity or viral gene expression, indicating that vIRF3-MT, which lacks PKM2 interaction, did not influence virus production and infectivity (Figs. 5b,c). Collectively, our results demonstrate that vIRF3–PKM2 mediated metabolic regulation does not affect KSHV life cycle.

To confirm the role of vIRF3–PKM2 interaction in the context of KSHV infection, we measured PK activity and acetyl-CoA level in LEC-E6/E7 cells infected with rKSHV-WT, rKSHV-vIRF3-MT, and rKSHV-REV-vIRF3-MT. PK activity and acetyl-CoA level were significantly reduced in LEC-E6/E7 cells infected with rKSHV-vIRF3-MT compared with LEC-E6/E7 infected with rKSHV-WT and rKSHV-REV-vIRF3-MT (Fig. 5d). To further investigate whether the vIRF3–PKM2 interaction is required for KSHV-mediated LEC invasiveness, we performed a 3D spheroid invasion assay on Matrigel using LEC-E6/E7 cells infected with rKSHV-BAC16-Flag-WT, rKSHV-BAC16-Flag-vIRF3-MT, or rKSHV-BAC16-Flag-REV-vIRF3-MT. Both WT and REV-vIRF3-MT infected cells showed marked invasion, whereas those infected with vIRF3-MT KSHV did not (Fig. 5e).

Given our findings in vIRF3-overexpressing LEC-E6/E7 cells, we next investigated whether vIRF3–PKM2-dependent metabolic reprogramming is also observed in KSHV-infected primary LECs. Consistent with our overexpression studies, infection with rKSHV-WT or rKSHV-REV robustly increased PK activity and intracellular acetyl-CoA levels, whereas infection with rKSHV-vIRF3-MT failed to induce comparable changes (Fig. 5f). Notably, because these experiments were performed in primary LECs, the observed metabolic reprogramming cannot be attributed to E6/E7-mediated immortalization and instead reflects a phenotype maintained during KSHV infection.

To further evaluate the contribution of the vIRF3–PKM2 interaction to KSHV-associated tumorigenic potential, we performed soft agar colony formation assays using primary LECs infected with rKSHV-BAC16-Flag-WT, rKSHV-vIRF3-MT, or rKSHV-REV (Fig. 5g). Primary LECs infected with rKSHV-WT or rKSHV-REV formed numerous well-defined colonies, whereas colony formation was markedly diminished in cells infected with rKSHV-vIRF3-MT. These findings suggest that disruption of the vIRF3–PKM2 interaction compromises KSHV-induced tumorigenic potential in primary LECs. Collectively, these results demonstrate that although the vIRF3–PKM2 interaction is dispensable for the KSHV life cycle, it is critical for vIRF3-driven metabolic reprogramming, EndMT, and KSHV-induced tumorigenic potential.

### vIRF3–PKM2-mediated upregulation of PKM2 activity promotes KSHV-associated tumor progression

**Fig. 6:**
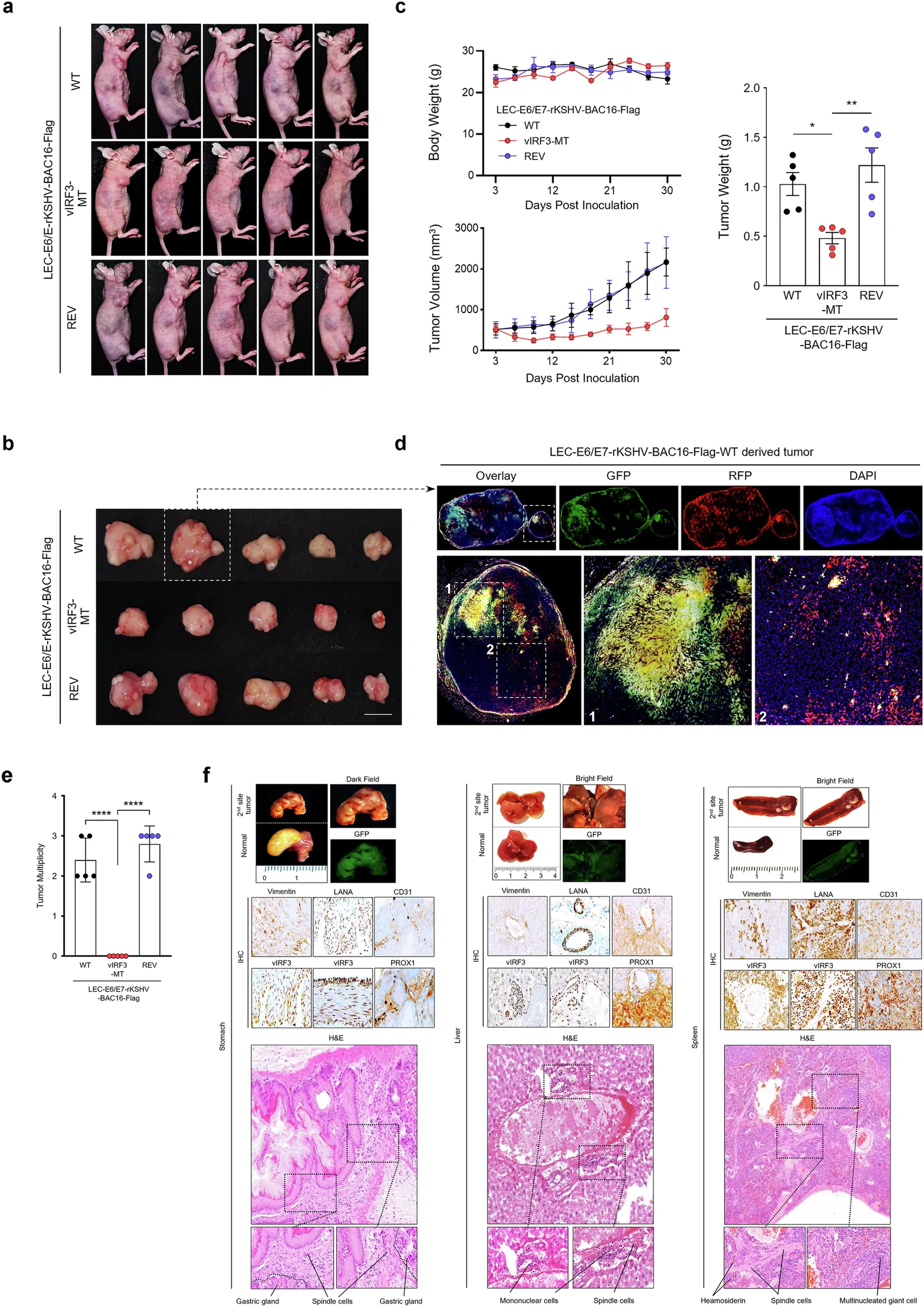
KSHV vIRF3–PKM2 interaction is required for KS-like tumor progression in xenograft model. **a** Representative photograph showing tumor growth in athymic Ncr-nu/nu mice at 30 days post-inoculations with LECs infected with rKSHV-BAC16-Flag-vIRF3-WT, rKSHV-BAC16-Flag-vIRF3-MT, or rKSHV-BAC16-Flag-REV-vIRF3-MT. **b** Representative images of dissected tumors from each xenograft group. **c** Tumor growth was monitored for 30 days. Graphs represent mean tumor volume (left), tumor weight at endpoint (middle), and mouse body weight over the course of the experiment (right). Data are presented as mean ± s.d., *n* = 5 mice per group. Statistical significance was assessed using one-way ANOVA; *P* < 0.05 was considered significant. *P < 0.05 and ***P < 0.0005. **d** Tumors from LEC-rKSHV-BAC16-Flag-vIRF3-WT xenograft mice were fixed and imaged under fluorescence microscopy with the indicated channels; DAPI was used for nuclear staining. **e** Quantification of secondary tumor multiplicity in each group. The number of secondary tumors per mouse is shown as mean ± s.d., *n* = 5 mice per group. Statistical significance was assessed using one-way ANOVA; *P* < 0.05 was considered significant. ****P < 0.0001. **f** Dissected organs from LEC-rKSHV-BAC16-Flag-vIRF3-WT xenograft mice were analyzed by immunohistochemistry using indicated antibodies; H&E staining served as a control staining.

Given that mesenchymal transition is associated with tumor growth and progression^43–45^, we conducted subcutaneous xenograft experiments using LECs infected with rKSHV-BAC16-Flag-WT, rKSHV-vIRF3-MT, or rKSHV-REV-vIRF3-MT to evaluate tumor development, volume, weight, and body weight changes. Both rKSHV-BAC16-Flag-WT and rKSHV-REV-vIRF3-MT efficiently promoted tumor initiation and growth, whereas rKSHV-BAC16-Flag-vIRF3-MT did not (Fig. 6a–c). These rKSHV-BAC16 constructs contain two distinct reporter genes between KSHV life cycle phases; GFP, driven by the EF1α promoter within the KSHV genome, represents latent infection, whereas RFP, driven by the KSHV PAN lytic promoter, indicates lytic cycle^20^. Tumors derived from LEC-E6/E7-rKSHV-BAC16-Flag-WT were harvested, embedded in paraffin block, stained with DAPI, and imaged for GFP and RFP fluorescence to assess KSHV life cycle. Interestingly, both latent and lytic programs were active within the tumor (Fig. 6d). Especially, regions with high lytic gene expression had lower cell density, whereas regions with robust latent gene expression exhibited higher cell density, as demonstrated by DAPI staining. These findings indicated that the bimodal expression of KSHV genes is crucial for KSHV pathogenesis.

Notably, GFP expression was detected in multiple organs, including the stomach, spleen, and liver, indicating the dissemination of KSHV-infected LECs to secondary sites beyond the inoculation site in mice injected with LEC-E6/E7-rKSHV-BAC16-Flag-WT or LEC-E6/E7-rKSHV-REV-vIRF3-MT (Fig. 6e,f and Table 1). In contrast, no secondary tumor formation was observed in mice injected with LEC-E6/E7-rKSHV-BAC16-Flag-vIRF3-MT. Histopathological examination of the secondary lesions revealed KS-like features, including extravascular erythrocytes, hemosiderin, and spindle-shaped cells (Fig. 6f). In the stomach, abnormal spindle cells exhibited proliferative and infiltrative growth. The spleen displayed atypical spindle cells along with multiple multinucleated giant cells around the trabecula. In the liver, mononuclear cell infiltrates and mild spindle cell proliferation were observed around the parenchymal vessels.

**Table 1:** Metastasis incidence on second-site organs of xenograft model.

rKSHV-BAC16	No.	Tumor Weight (g)	Organ	GFP Signal	Morphological Abnormality
**Flag-vIRF3** **-WT**	**1**	1.11	Stomach	++	Malformation
			Spleen	+	Enlarged
			Kidney	N.D.	Tumor nodules
	**2**	1.12	Stomach	++	N.D.
			Spleen	+	Enlarged
	**3**	0.79	Liver	+	N.D.
			Stomach	++	Malformation
			Spleen	+	N.D.
			Kidney	N.D.	Tumor nodule, enlarged
			Heart	N.D.	Malformation, bleeding
	**4**	0.45	Stomach	++	N.D.
			Spleen	+	Enlarged
			Lymph node	+++	N.D.
	**5**	0.34	Stomach	++	Malformation, bleeding
			Spleen	+	Enlarged, tumor nodule
			Heart	N.D.	Shrunken, bleeding
**Flag-vIRF3** **-MT**	**6**	0.58	N.D.	N.D.	
	**7**	0.59	N.D.	N.D.	
	**8**	0.55	N.D.	N.D.	
	**9**	0.38	N.D.	N.D.	
	**10**	0.07	N.D.	N.D.	
**Flag-vIRF3** **-REV**	**11**	1.19	Stomach	++	Malformation
			Spleen	+	N.D.
			Lymph node	++	Enlarged
	**12**	1.47	Stomach	++	N.D.
			Spleen	+	Enlarged
			Cecum	+	Inflated
	**13**	0.58	Stomach	++++	N.D.
			Spleen	+	N.D.
	**14**	0.37	Stomach	+	N.D.
			Spleen	+	Enlarged
			Kidney	N.D.	Enlarged
	**15**	0.41	Stomach	+	N.D.
			Spleen	+	Enlarged
			Liver	+	Shrunken

Consistent with the KS-like morphologic features, immunohistochemical analysis revealed that spindle cells and infiltrating atypical cells within the secondary lesions were positive for the KSHV latency-associated nuclear antigen (LANA) and vIRF3 (Fig. 6f). To further define their cellular identity, we examined the expression of the vascular endothelial marker CD31 and the lymphatic endothelial lineage-specific transcription factor PROX1. Both markers were readily detected in spindle cells and atypical cells within the secondary lesions of the stomach, spleen, and liver. Importantly, the co-expression of CD31 and PROX1 together with KSHV-associated viral antigens (LANA and vIRF3) supports the presence of a dual endothelial lineage phenotype, a hallmark feature of KS. These findings indicate that the disseminated lesions retain key phenotypic characteristics of KS rather than representing undifferentiated sarcomas. Importantly, human vimentin-positive cells, derived from KSHV-infected LECs, were identified in the secondary tumor sites (Fig. 6f). These results suggest that the proliferation of KSHV-positive or atypical cells observed in the stomach, liver, and spleen was derived from implanted KSHV-human endothelial cells. Taken together, our results highlight the pivotal role of the vIRF3–PKM2 interaction in promoting tumor progression through EndMT-driven migration.

### vIRF3-derived peptide leading to a marked regression of KSHV-associated tumor

**Fig. 7:**
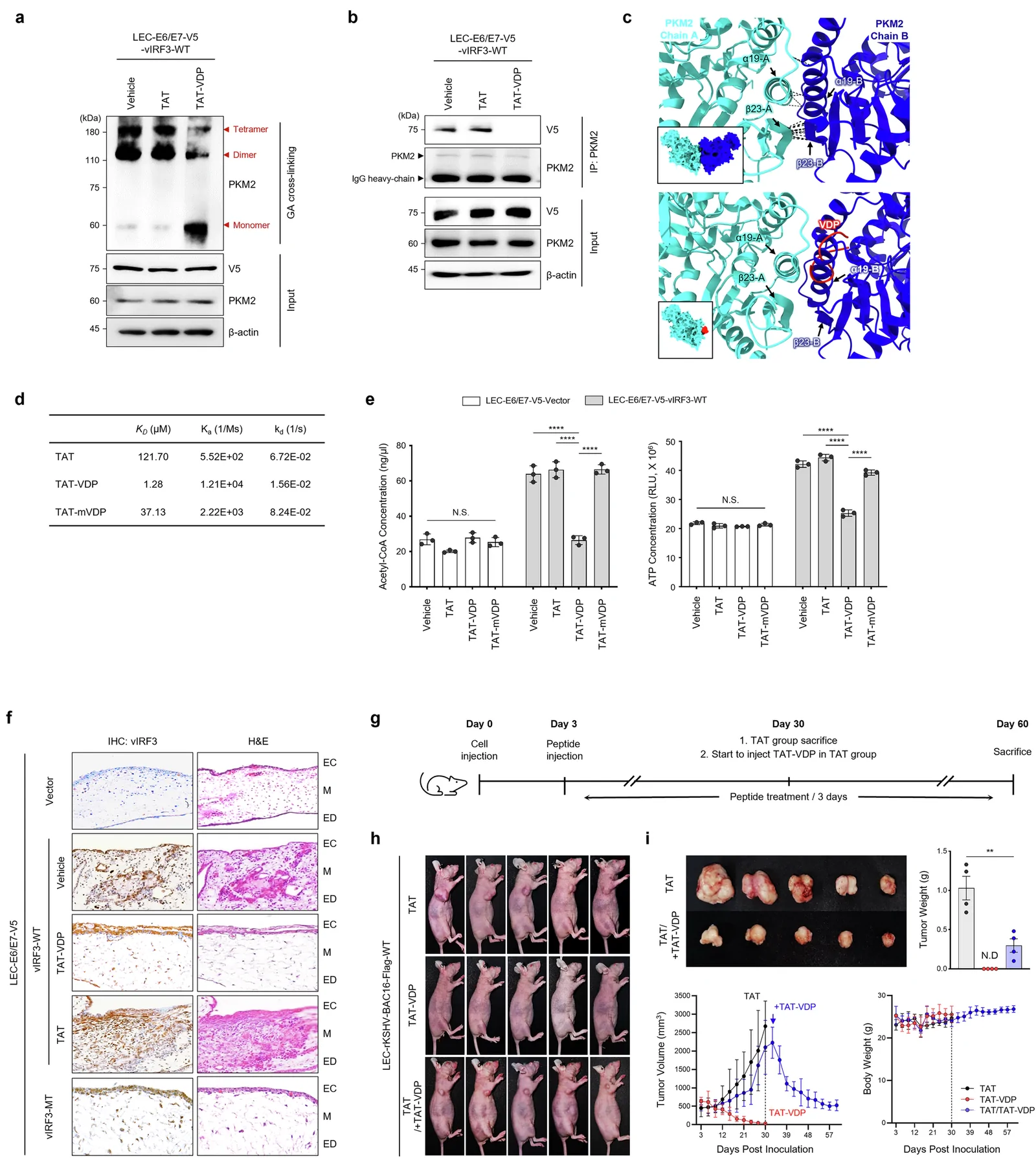
KSHV vIRF3-derived peptide competitively inhibits the vIRF3–PKM2 interaction, thereby inhibiting KS-like tumor progression. **a** LEC-E6/E7-V5-vIRF3-WT cells treated with vehicle, TAT, or TAT-VDP were lysed and analyzed for PKM2 multimerization using 0.15% glutaraldehyde. IB was performed on 4–12% gradient SDS–PAGE using the indicated antibodies. **b** LEC-E6/E7-V5-vIRF3-WT cells were incubated with vehicle, TAT, or TAT-VDP, then IP with anti-PKM2, and followed by IB with anti-V5 and anti-PKM2 antibodies. **c** Depiction of subunit contacts between chains A and B of PKM2 (PDB ID: 1ZJH). Intersubunit interactions are indicated by dotted lines, and secondary structures of mPKM2 involved in the subunit contacts labelled (upper). The predicted binding mode of VDP to PKM2 overlapped with the structures of PKM2 chains A and B. Secondary structures of monomeric PKM2 involved in VDP binding are labelled (lower). **d** mVDP represents a mutant VDP that potentially loses its interaction with PKM2. BLI kinetic assay was performed with VDP or mVDP against purified PKM2 protein in vitro. **e** LEC-E6/E7-V5-vector or LEC-E6/E7-V5-vIRF3 were treated with vehicle, TAT, TAT-VDP, or TAT-mVDP, then harvested to measure cellular acetyl-CoA and ATP levels. Data are presented as mean ± s.d., *n* = 3 independent biological experiments. Statistical significance was assessed using one-way ANOVA; *P* < 0.05 was considered significant. N.S. non-significant. *P* > 0.05, *P < 0.05, ****P < 0.0001. **f** LEC-E6/E7-V5-vector, LEC-E6/E7-V5-vIRF3-WT, or LEC-E6/E7-V5-vIRF3-MT cells were seeded onto the CAM of 10-day-old chick embryos and treated with vehicle, TAT, or TAT-VDP. On day 14, CAMs were harvested, fixed, and analyzed by immunohistochemical staining using a vIRF3 antibody. H&E staining served as a control. EC ectoderm, ED endoderm, M mesoderm. **g** Schematic of peptide treatment in Ncr-nu/nu mice bearing tumors derived from LEC-rKSHV-BAC16-Flag-vIRF3-WT cells. **h** LEC-rKSHV-BAC16-Flag-vIRF3-WT cells were injected subcutaneously into Ncr-nu/nu mice, followed by treatment with either 0.2 mg of TAT, TAT-VDP, or TAT-VDP after TAT treatment. **i** Dissected tumors and tumor weight were measured after killing the mice (upper). Mean tumor volume and body weight were monitored through 60 days (lower). Black dot, TAT-treated group; red dot, TAT-VDP-treated group; blue dot, TAT-VDP-treated group after 30 days of TAT treatment. Data are presented as mean ± s.d., *n* = 5 mice per group. Statistical significance was assessed using one-way ANOVA; *P* < 0.05 was considered significant. **P < 0.005, N.D. non-detectable.

Series of binding assays identified the vIRF3 region (aa250-260) as sufficient for its interaction with PKM2 (Supplementary Fig. 5a,b), leading to the development of a vIRF3-derived peptide, termed ‘VDP’. To examine the effect of VDP on PK activity and the allosteric state of PKM2, we fused it with the HIV1 TAT transduction domain for intracellular delivery^46,47^. LEC-E6/E7-V5-vIRF3-WT cells were treated with TAT or TAT-VDP, followed by a PK activity assay or cross-linking with glutaraldehyde. VDP effectively inhibited vIRF3-induced PK activity and vIRF3-mediated tetramer formation of PKM2 (Fig. 7a and Supplementary Fig. 5c). To determine whether VDP inhibits vIRF3-mediated tetramer formation of PKM2 by competing for PKM2 binding, we treated LEC-V5-vIRF3-WT cells with TAT or TAT-VDP and performed IP using an anti-PKM2 antibody. TAT-VDP treatment effectively blocked vIRF3 binding to PKM2, whereas TAT or vehicle treatment maintained the interaction intact (Fig. 7b). To investigate how VDP inhibits PKM2 tetramerization at the structural level, we used AlphaFold multimer to model VDP bound to the monomeric PKM2 (mPKM2). Among the predicted structures, model 3, which interacts with α19-A and β23-A of mPKM2, showed the lowest interface energy, primarily because of hydrophobic interactions between the α-helices (α19-A: α19-B) and β-strands (β23-A: β23-B) of adjacent subunits (Fig. 7c, Supplementary Fig. 5d–g, and Supplementary Table 1). To further validate the binding affinity of VDP for PKM2, we performed an in vitro binding assay comparing VDP with its mutant counterpart mVDP, which was designed by mutating residues involved in PKM2 binding. VDP exhibited a high binding affinity for PKM2, with a dissociation constant (*K*_d_) of 1.28 µM, whereas mVDP showed a 29-fold lower binding affinity (Fig. 7d and Supplementary Fig. 5h). Importantly, mVDP failed to suppress vIRF3-mediated upregulation of acetyl-CoA and ATP level, unlike VDP (Fig. 7e). Overall, these findings demonstrate that VDP peptide strongly binds to PKM2, effectively inhibiting vIRF3-mediated PKM2 function by competing with vIRF3 for PKM2 binding.

To assess the anti-invasive potential of VDP, LEC-V5-vector, LEC-V5-vIRF3-WT, or LEC-V5-vIRF3-MT cells were seeded on the chick embryo CAM. After overnight incubation, we treated the CAM with either TAT or TAT-VDP, followed by immunohistochemical analysis. The CAM consists of three germ layers: ectoderm, mesoderm, and endoderm^48,49^. LEC-V5-vIRF3-WT cells invaded deep into the endoderm layer, whereas LEC-vector and LEC-vIRF3-MT remained in the ectoderm (Fig. 7f). Notably, TAT-VDP treatment significantly reduced the vIRF3-mediated invasion compared with TAT treatment, indicating that VDP effectively suppressed invasion by vIRF3–PKM2-associated EndMT (Fig. 7f). To further evaluate the anti-tumor effects of VDP, we used NCr-nu/nu xenograft mice subcutaneously injected with rKSHV-BAC16-Flag-WT-infected LECs. Mice received 0.2 mg (equivalent to 25 μM) of each peptide twice per week for 30 days (Fig. 7g). TAT-VDP treatment markedly inhibited tumor formation and significantly reduced tumor size in advanced tumors cases compared with TAT treatment (Fig. 7h,i) without any substantial toxicity (Supplementary Fig. 5i–k). Collectively, these findings elucidate the in vivo anti-tumor effect of the VDP peptide in suppressing tumor development and metastasis.

## Discussion

The dissemination of KSHV-infected cells in the visceral organs of KS patients is linked to poor prognoses. However, classifying KS as a conventional metastatic cancer is challenging because of its multifocal distribution across various tissue areas and the lack of direct evidence regarding KSHV-driven tumor metastasis^50–53^. Previous study has attempted to assess KS clonality by analyzing the methylation pattern of the X-linked HUMARA in multiple lesions from the same patient with KS, supporting the notion that KS is a disseminated monoclonal cancer^52^. Moreover, KS had higher levels of endothelial progenitor cells in peripheral blood, which are KSHV positive and capable of maintaining viral infection^54,55^. Here we provide novel mechanistic insights into KSHV-associated tumor progression by identifying vIRF3-mediated metabolic reprogramming in LECs. Collectively, the vIRF3–PKM2 interaction leads to EndMT by upregulating acetyl-CoA levels, promoting LEC invasion and migration. More importantly, this process induces secondary tumorigenesis in the visceral organs of xenograft mice, closely resembling the clinical characteristics of KS in human, recapitulating the histopathological features of disseminated KS. Notably, targeting PKM2 using a vIRF3-derived peptide led to the regression of KS-like tumor in xenograft mouse models, indicating that PKM2 is a potential novel target for anti-cancer therapeutics for KS. These findings highlight the critical role of vIRF3-driven acetyl-CoA flux in LECs during the KS progression. Nevertheless, the vIRF3–PKM2–acetyl-CoA–SMAD5 axis remains to be validated in patient-derived KS tissues, owing in part to the limited availability of appropriately preserved and clinically annotated specimens in our region^56^. Future analyses of vIRF3, PKM2, and acetylated SMAD5 in clinical KS samples spanning different stages of disease progression will be important to determine the clinical relevance of this pathway. Finally, although our xenograft model recapitulates dissemination-associated KS-like lesions in visceral organs, elucidating the precise trafficking route by which tumor cells reach these sites was beyond the scope of the present study. Accordingly, the present data do not distinguish bona fide metastatic dissemination from alternative mechanisms of systemic distribution. This question will require further investigation in future studies.

The classic example of a reprogrammed metabolic pathway in cancer is the Warburg effect, also known as aerobic glycolysis. However, growing evidence underscores the crucial role of metabolic flexibility at every stage of cancer progression^2,57,58^. In particular, TCA cycle-derived metabolites have re-emerged as key regulators of tumor growth and metastasis, modulating various signaling pathways in both cancer mouse models and human patients^2^. Both glycolysis and the TCA cycle play essential roles in tumor progression by supporting metabolite biosynthesis. Accordingly, recent findings highlight the metabolic flexibility required at different stages of KSHV replication and pathogenesis. For instance, Sanchez et al. demonstrated that KSHV-latently infected TIME cells rely on glutamine-mediated replenishment of the TCA cycle (anaplerosis) for cell survival^18^. Furthermore, Ying et al. reported that vFLIP suppresses aerobic glycolysis in an NF-κB-dependent manner to orchestrate the tumor stress microenvironment^19^. More importantly, most studies on the Warburg effect in the context of KSHV have focused on hypoxic conditions in KSHV-infected cells rather than on the broader metabolic landscape of KS, leaving a gap in understanding how metabolic reprogramming contributes to KS progression. In addition, despite the crucial role of TCA cycle-derived metabolites in the metastatic stage, there is currently not much evidence directly linking viral genes to the regulation of host metabolic enzymes involved in the TCA cycle to regulate tumor progression. Here we demonstrated that vIRF3 specifically interacts with PKM2 (Fig. 1 and Supplementary Fig. 1), enhancing its enzymatic activity by promoting active allosteric conformation (Figs. 2 and 3). This elevation of PK activity in cells induces acetyl-CoA to activate the TCA cycle instead of aerobic glycolysis. Moreover, our isotope tracing with [U-^13^C] glucose confirms the upregulation of glutamate levels derived from α-KG, suggesting vIRF3 did not rely on glutaminolysis to generate TCA cycle metabolites, which differs from other cancers^59^. Notably, vIRF3-mediated accumulation of acetyl-CoA as the primary TCA cycle intermediate occurs without replenishment from anaplerosis. Interestingly, unlike the regulation of aerobic glycolysis by KSHV, these effects contribute to KS tumor progression via EndMT rather than affecting the viral life cycle and virus production, highlighting the pivotal role of vIRF3 as a viral metabolic modulator using metabolic enzyme PKM2 to activate signaling metabolites that participate in the TCA cycle for KS metastasis.

In the present study, vIRF3 triggers PKM2, promoting pyruvate catabolism to fuel the TCA cycle, thereby increasing intracellular acetyl-CoA levels and ultimately facilitating the acetylation of the transcriptional factors SMAD5. These findings uncover a previously undescribed metabolic mechanism underlying EndMT regulation. Among the candidates identified in our acetylome profiling analysis, SMAD5 was prioritized for further mechanistic investigation because it emerged as one of the top acetylation candidates and has a well-established role in EndMT, consistent with the spindle-like phenotype observed specifically in vIRF3-expressing LECs. In addition to the primary vIRF3–PKM2–acetyl-CoA–SMAD5 axis, our screening analysis identified several other candidate genes, including CHAT, GTF2A1L, Tenascin C, and RHOXF2. Although these genes are not readily interpreted as direct regulators of EndMT in LECs, they may reflect broader transcriptional or microenvironment-associated changes induced by KSHV infection. For example, Tenascin C (ECM/matricellular protein) may represent a potential extracellular matrix-associated modifier of the tumor microenvironment, whereas CHAT (choline acetyltransferase), GTF2A1L (germ cell-associated transcriptional regulator), and RHOXF2 (reproductive/germline-associated transcription factor) are more likely to reflect context-dependent transcriptional response or secondary molecular changes associated with KSHV infection^60–62^. Further studies will be required to determine whether these candidates contribute indirectly to endothelial reprogramming or cooperate with the vIRF3-driven pathway described here. Indeed, acetyl-CoA levels influence cancer, immune system, and stem cell functions^1^. Thus, to enhance their ability to initiate and progress cancer, cancer cells upregulate enzymes involved in producing acetyl-CoA, such as ACLY. In this context, the inhibition of ACLY reportedly suppresses tumorigenesis^63^. Here we reported that enhancing basal acetyl-CoA may potentially occur through the catalysis of exported mitochondrial citrate, originating from pyruvate catabolism, by ACLY (Fig. 4a). These findings align with Fig. 3d, indicating that vIRF3, despite upregulating carnitine, reduces trans-2-enoyl-CoA and ketoacyl-CoA levels due to diminishing expression of CPT1A. This indicates that KSHV vIRF3 favors the pyruvate catabolism pathway rather than the FAO pathway to modulate acetyl-CoA abundance in LECs. Notably, our study also provides compelling evidence linking the aberrant accumulation of acetyl-CoA to its role as a substrate for the induction of protein acetylation, contributing to KSHV-associated metastasis. Although LEC-E6/E7 cells were used as the principal experimental platform because they provide a stable and reproducible system for mechanistic analyses, the key metabolic and transformation-associated phenotypes were also validated in primary human LECs infected with recombinant KSHV (Fig. 5f,g). Thus, LEC-E6/E7 should be viewed primarily as a technical tool for experimental standardization and reproducibility rather than as a biologically essential component of the mechanism described here.

In summary, our study provides experimental evidence elucidating the role of the vIRF3–PKM2 axis in governing acetylated SMAD5-mediated EndMT by upregulation of post-translational modification, acetyl-CoA, thereby contributing to the metastatic progression of disseminated KS. Notably, vIRF3-derived short VDP peptide efficiently competes with vIRF3–PKM2 interaction, effectively suppressing acetyl-CoA levels together with PK activity in KSHV-infected cells and suppressing tumor progression in a mouse xenograft model. Of special importance, we herein report that the VDP peptide represents a potential novel chemotherapeutic molecule that specifically targets metastatic KS therapies.

### Supplementary information

Supplementary information accompanies the manuscript on the Experimental & Molecular Medicine website (http://www.nature.com/emm/).

## Supplementary information

Supplementary Information

Western Blot Raw Data 1

Western Blot Raw Data 2

Western Blot Raw Data 3

Western Blot Raw Data 4

Western Blot Raw Data 5

Western Blot Raw Data 6

Western Blot Raw Data 7

Western Blot Raw Data 8

Western Blot Raw Data 9

Western Blot Raw Data 10

## Data Availability

The mass spectrometry data for binding partner screening is available on PRIDE website with accession number of PXD053617 (http://www.ebi.ac.uk/pride/). Metabolomics data is available on MetaboLights website with identifier number of MTBLS10708 (http://www.ebi.ac.uk/metabolights/MTBLS10708). All other data are available in the main text or the Supplementary Information.
