# Supplementary material for: KSHV vIRF3–PKM2 interaction induces SMAD5 acetylation, promoting Kaposi’s sarcoma progression
Source: Exp Mol Med. 2026 Oct 5;58(10):3423–39. doi: 10.1038/s12276-026-01849-2 (PMC13737100; doi:10.1038/s12276-026-01849-2)
Supplement: Supplementary file 1 [file 12276_2026_1849_MOESM1_ESM.pdf]

Supplementary Information

KSHV vIRF3-PKM2 Interaction Induces SMAD5 Acetylation, Promoting Kaposi's Sarcoma Progression

Yeong Jun Kim[1], Juhyeon Lim[2], Jae Jin Lee[2], Abhishek Kumar[3], Shrute Kannappan[4], Junjie Zhang[5], Soochi Kim[1], Han-Jun Kim[6], Kyeong Kyu Kim[4], Zsolt Toth[3], Hyungjin Eoh[2,7], Kyung-Chul Choi[8]*, and Hye-Ra Lee[1,9]*

[1]Department of Biotechnology and Bioinformatics, College of Science and Technology, Korea University; Sejong, 30019, Republic of Korea
[2]Department of Molecular Microbiology and Immunology, University of Southern California; Los Angeles, CA, 90033, USA
[3]Department of Oral Biology, College of Dentistry, University of Florida; Gainesville, FL, 32610, USA
[4]Department of Precision Medicine, Sungkyunkwan University School of Medicine; Suwon, 16419, Republic of Korea
[5]The State Key Laboratory of Virology, Medical Research Institute, Wuhan University; Wuhan, China
[6]College of Pharmacy, Korea University; Sejong, 30019, Republic of Korea
[7]Department of Ophthalmology, University of Southern California; Los Angeles, CA, 90033, USA
[8]Department of Biomedical Sciences, Asan Medical Center, University of Ulsan College of Medicine, Seoul, 05505, Republic of Korea
[9]Department of Lab Medicine, College of Medicine, Korea University; Seoul, 08308, Republic of Korea

Running title: Induction of disseminated KS by KSHV vIRF3-PKM2 interaction

*Co-Correspondence authors: Kyung-Chul Choi and Hye-Ra Lee

Kyung-Chul Choi
Department of Biomedical Science
Asan Medical Center, University of Ulsan College of Medicine,
Seoul 05505, Republic of Korea.
Phone: (+82) 2-2010-2087
Fax: (+82) 2-3010-2642
E-mail: choikc75@amc.seoul.kr

Hye-Ra Lee
Department of Biotechnology and Bioinformatics
College of Science and Technology, Korea University
2511 Sejong-ro, Sejong, 30019, Republic of Korea
Phone (+82) 44-860-1831
Fax (+82) 44-860-1598
Email: leehr@korea.ac.kr

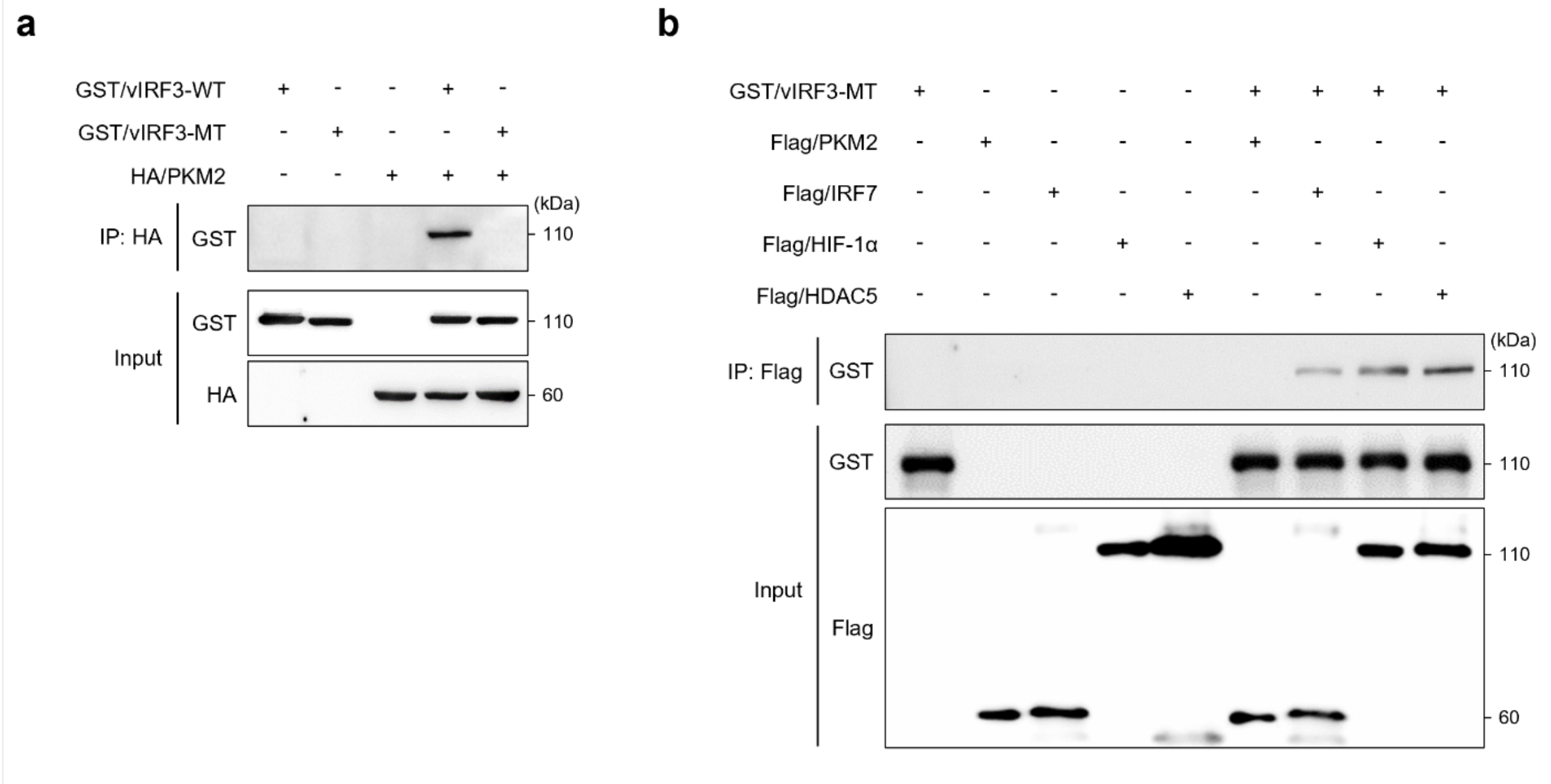

Supplementary Fig. 1 a 293T cells were transfected with PKM2 and vIRF3 wild-type (vIRF3-WT) or PKM2-interaction deficient mutant of vIRF3 (vIRF3-MT). Then immunoprecipitation was performed with anti-HA antibody followed by immunoblotting (IB) with anti-GST antibody. **b** vIRF3-MT was co-transfected into 293T cells along with known-interaction partner proteins of vIRF3. Cell lysate was immunoprecipitated (IP) with anti-Flag antibody followed by IB using anti-GST antibody.

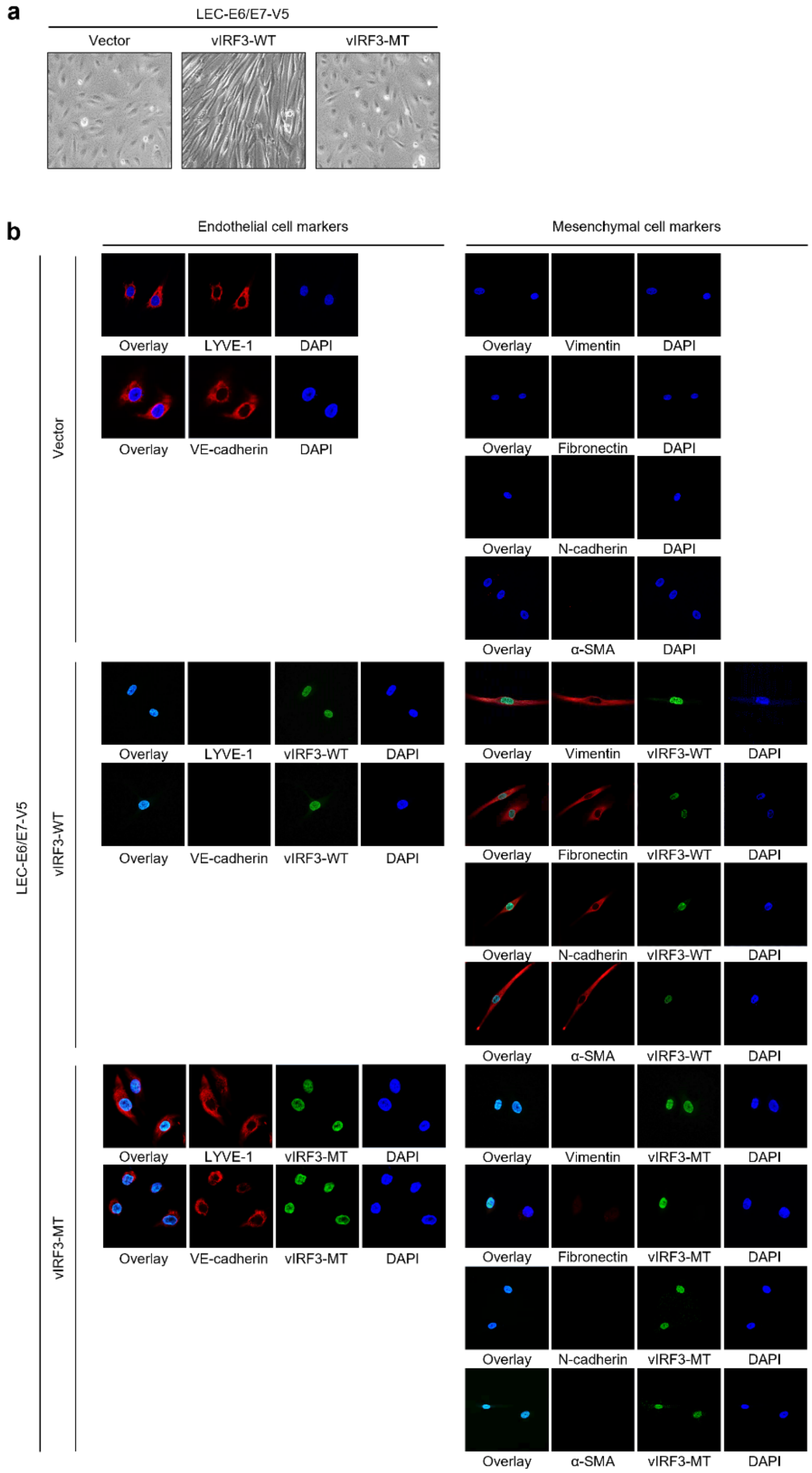
a
LEC-E6/E7-V5
Vector
vIRF3-WT
vIRF3-MT
b
Endothelial cell markers
Mesenchymal cell markers
LEC-E6/E7-V5
Vector
vIRF3-WT
vIRF3-MT
Overlay
LYVE-1
VE-cadherin
DAPI
Vimentin
Fibronectin
N-cadherin
α-SMA
vIRF3-WT
vIRF3-MT

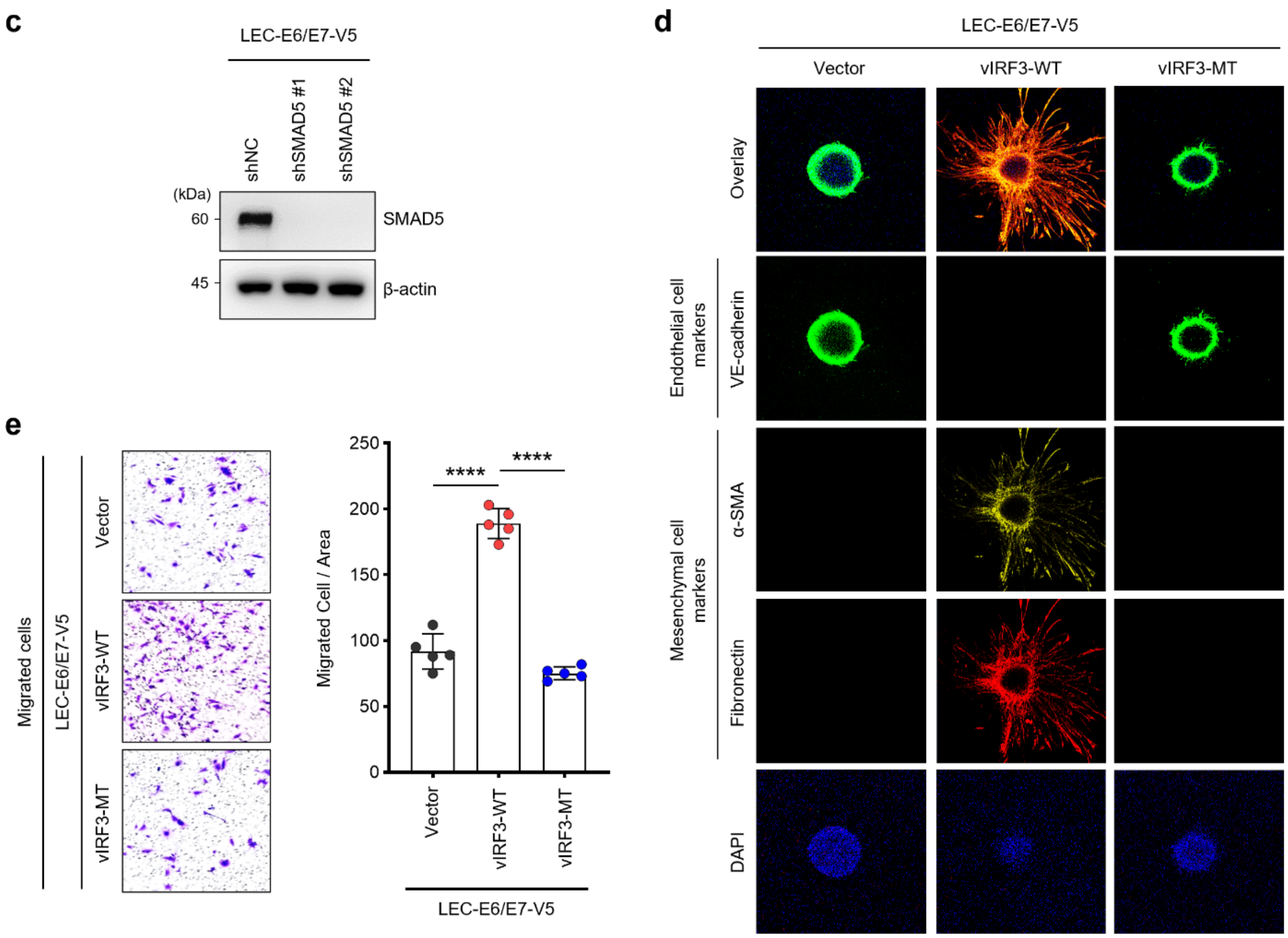

Supplementary Fig. 2 **a** The representative images of LEC-E6/E7-V5-vector, LEC-E6/E7-V5-vIRF3-WT, and LEC-E6/E7-V5-vIRF3-MT. **b** LECs stably expressing vector, vIRF3-WT, and vIRF3-MT were seeded on the glass disk for immunostaining with antibody targeting indicated endothelial or mesenchymal-specific marker proteins. The nuclei of the cells were stained with DAPI. **c** LEC-E6/E7 cells were transduced with lentivirus harboring SMAD5 shRNA (shSMAD5) or negative control shRNA (shNC). 24 hours after the transduction, cells were lysed for IB with an anti-SMAD5 antibody. **d** LEC-E6/E7 stably bearing vector, vIRF3-WT, vIRF3-MT were cultured for spheroid formation. Spheroids were seeded in the 3D Matrigel matrix and stained with indicated endothelial or mesenchymal marker antibody. **e** Migration capacity of LEC-E6/E7-V5-vector, LEC-E6/E7-V5-vIRF3-WT, and LEC-E6/E7-V5-vIRF3-MT were analyzed by Boyden's chamber assay using 8 μm-pore transwell insert.

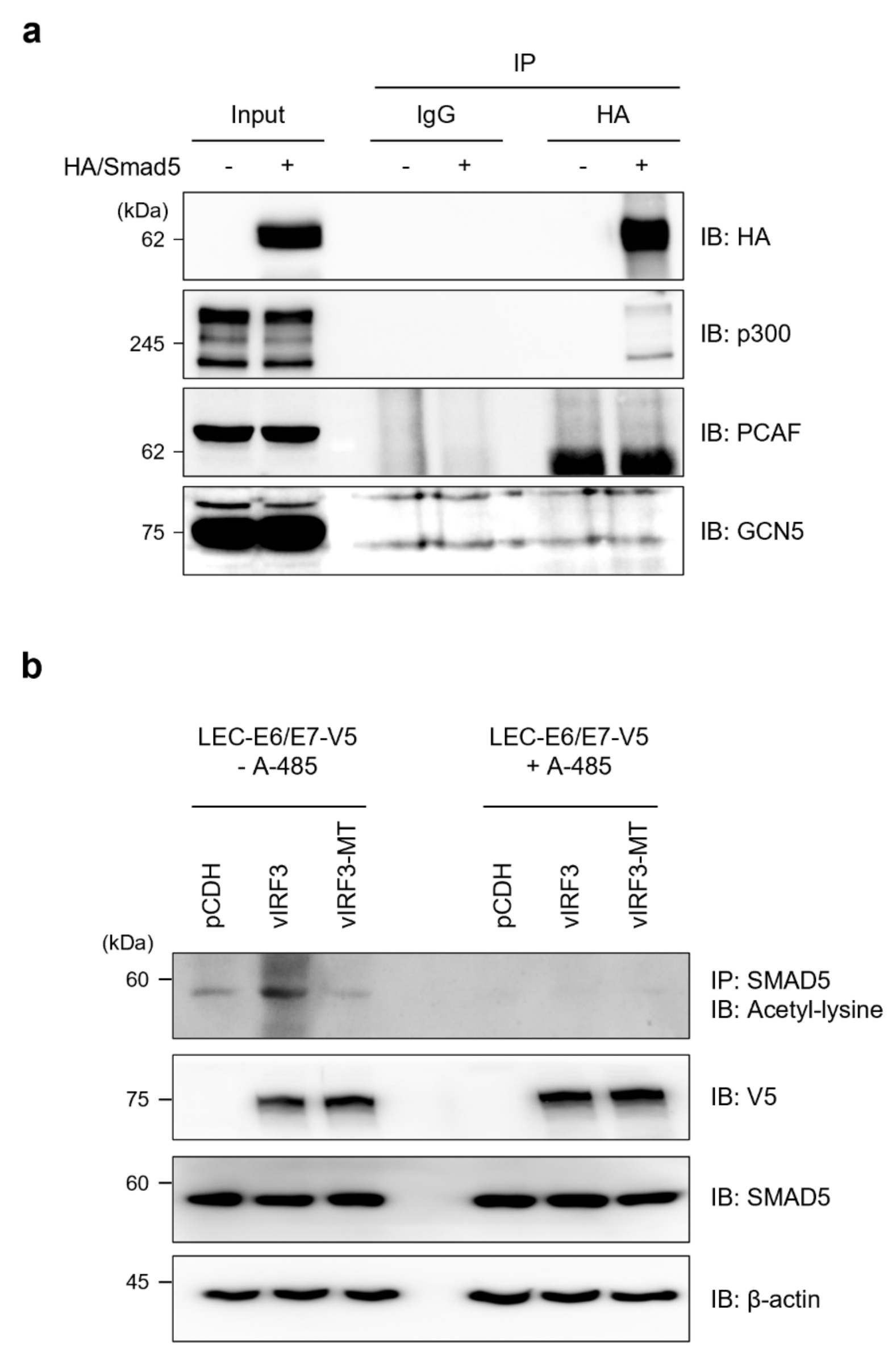

Supplementary Fig. 3 a Interaction of SMAD5 with representative KATs. HEK293T cells were transfected with HA-tagged SMAD5, followed by IP using an anti-HA antibody or normal IgG as a negative control. Co-IP proteins were analyzed by IB with antibodies against p300, PCAF, and GCN5. **b** Effect of p300 inhibition on SMAD5 acetylation. LEC-E6/E7-V5 cells expressing vector (pCDH), vIRF3, or vIRF3-MT were treated with 5 μM of selective p300 inhibitor A-485. SMAD5 was immunoprecipitated using an anti-SMAD antibody and acetylation levels were determined by IB using an anti-acetyl-lysine antibody.

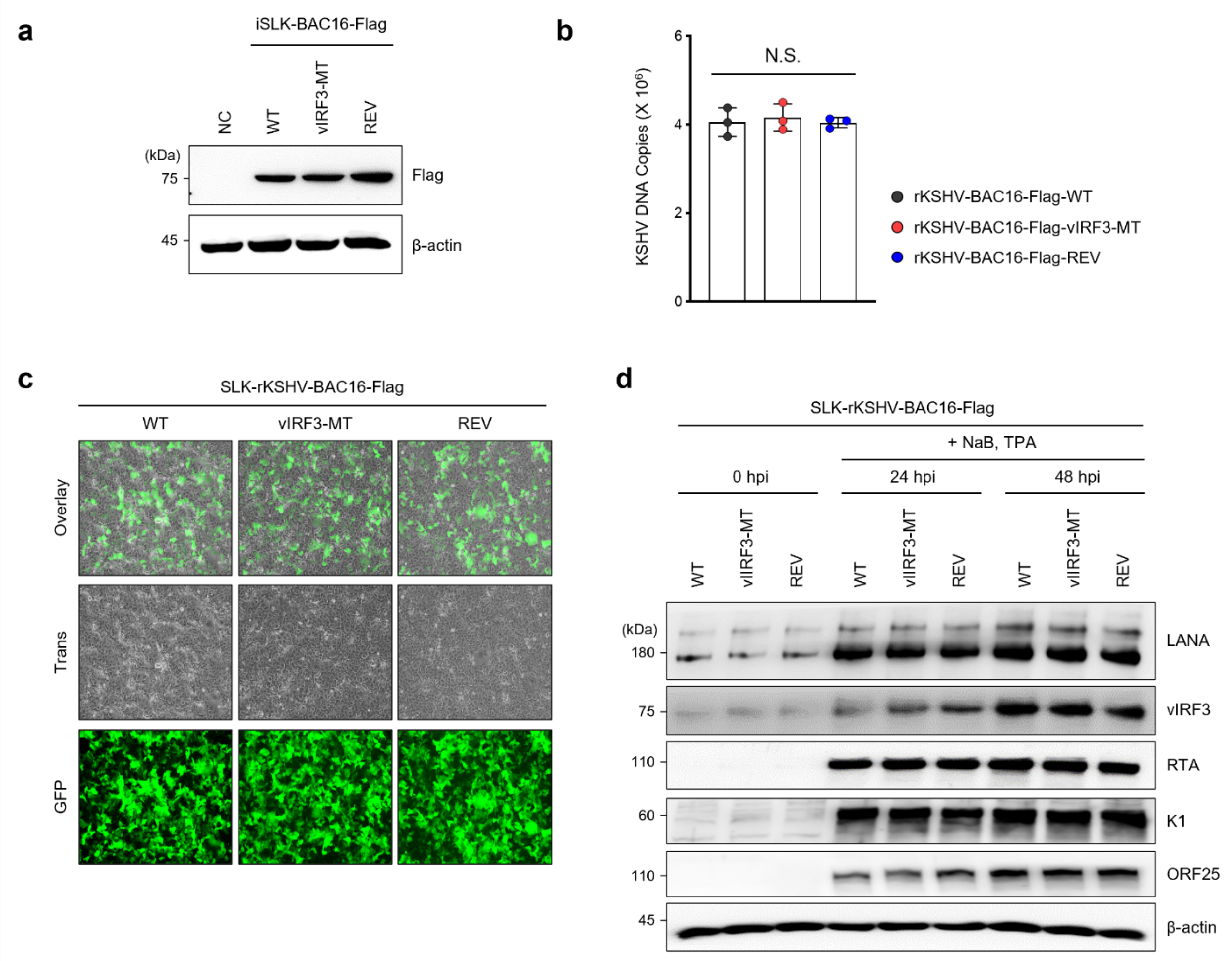

Supplementary Fig. 4 a The lysate from iSLK stably expressing BAC16-Flag-vIRF3-WT, BAC16-Flag-vIRF3-MT, and BAC16-Flag-REV were IB with anti-Flag antibody. **b** iSLK cells stably carrying BAC16-Flag-vIRF3-WT, BAC16-Flag-vIRF3-MT, or BAC16-Flag-REV were induced with NaB and Doxycycline. The produced rKSHV was titrated by RT-PCR. N.S.; non-significant, p>0.05 **c** SLK cells were infected with rKSHV-BAC16-Flag-WT, rKSHV-BAC16-Flag-vIRF3-MT, or rKSHV-BAC16-Flag-REV and GFP expression was visualized at 72 hours post infection. **d** SLK cells infected with indicated rKSHV and induced using NaB and doxycycline at 72 hpi. After 48 hours of induction, cells were harvested, lysed for IB using indicated antibodies targeting viral proteins.

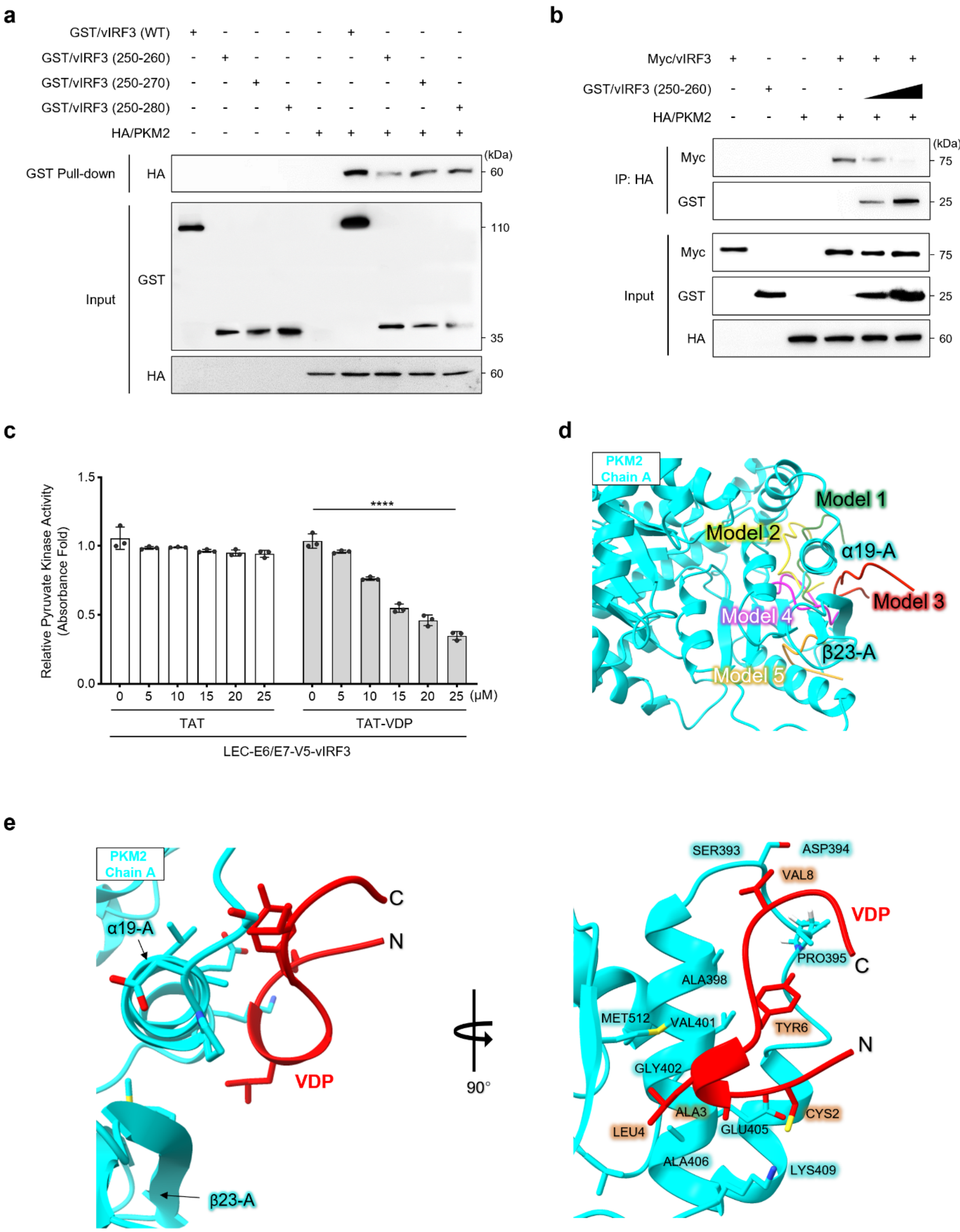
a
GST/vIRF3 (WT)
GST/vIRF3 (250-260)
GST/vIRF3 (250-270)
GST/vIRF3 (250-280)
HA/PKM2
GST Pull-down
HA
Input
GST
HA
(kDa)
60
110
35
60
b
Myc/vIRF3
GST/vIRF3 (250-260)
HA/PKM2
IP: HA
Myc
GST
Input
Myc
GST
HA
(kDa)
75
25
75
25
60
c
Relative Pyruvate Kinase Activity (Absorbance Fold)
1.5
1.0
0.5
0.0
****
0 5 10 15 20 25 0 5 10 15 20 25 (μM)
TAT
TAT-VDP
LEC-E6/E7-V5-vIRF3
d
PKM2 Chain A
Model 1
Model 2
α19-A
Model 3
Model 4
β23-A
Model 5
e
PKM2 Chain A
α19-A
C
N
VDP
β23-A
90°
SER393
ASP394
VAL8
VDP
PRO395
C
ALA398
MET512
VAL401
TYR6
N
GLY402
ALA3
CYS2
LEU4
GLU405
ALA406
LYS409

f

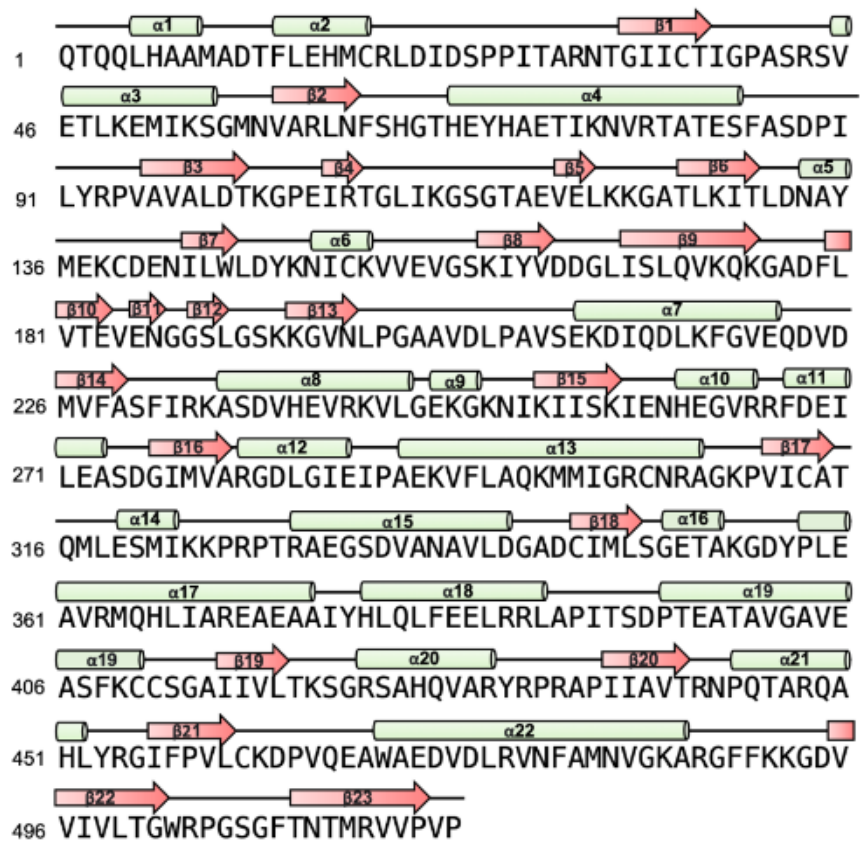
1 QTQQLHAAMADTFLEHMCRLDIDSPPITARNTGIICTIGPASRSV
46 ETLKEMIKSGMNVARLNFSHGTHEYHAETIKNVRTATESFASDPI
91 LYRPVAVALDTKGPEIRTGLIKGSGTAEVELKKGATLKITLDNAY
136 MEKCDENILWLDYKNICKVVEVGSKIYVDDGLISLQVKQKGADFL
181 VTEVENGGSLGSKKGVNLPGAAVDLPAVSEKDIQDLKFGVEQDVD
226 MVFASFIRKASDVHEVRKVLGEKGKNIKIISKIENHEGVRRFDEI
271 LEASDGIMVARGDLGIEIPAEKVFLAQKMMIGRCNRAGKPVICAT
316 QMLESMIKKPRPTRAEGSDVANAVLDGADCIMLSGETAKGDYPLE
361 AVRMQHLIAREAEAAIYHLQLFEELRRLAPITSDPTEATAVGAVE
406 ASFKCCSGAIIVLTKSGRSAHQVARYRPRAPIIAVTRNPQTARQA
451 HLYRGIFPVLCKDPVQEAWAEDVDLRVNFAMNVGKARGFFKKGDV
496 VIVLTGWRPGSGFTNTMRVVPVP

g

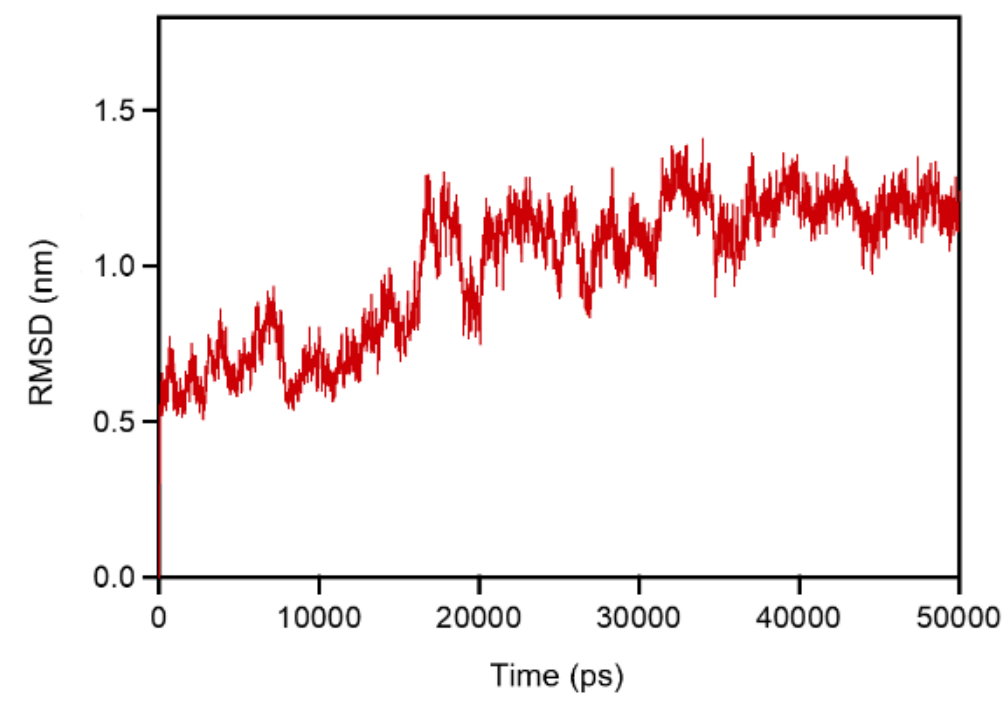
RMSD (nm)
0.0
0.5
1.0
1.5
0
10000
20000
30000
40000
50000
Time (ps)

h

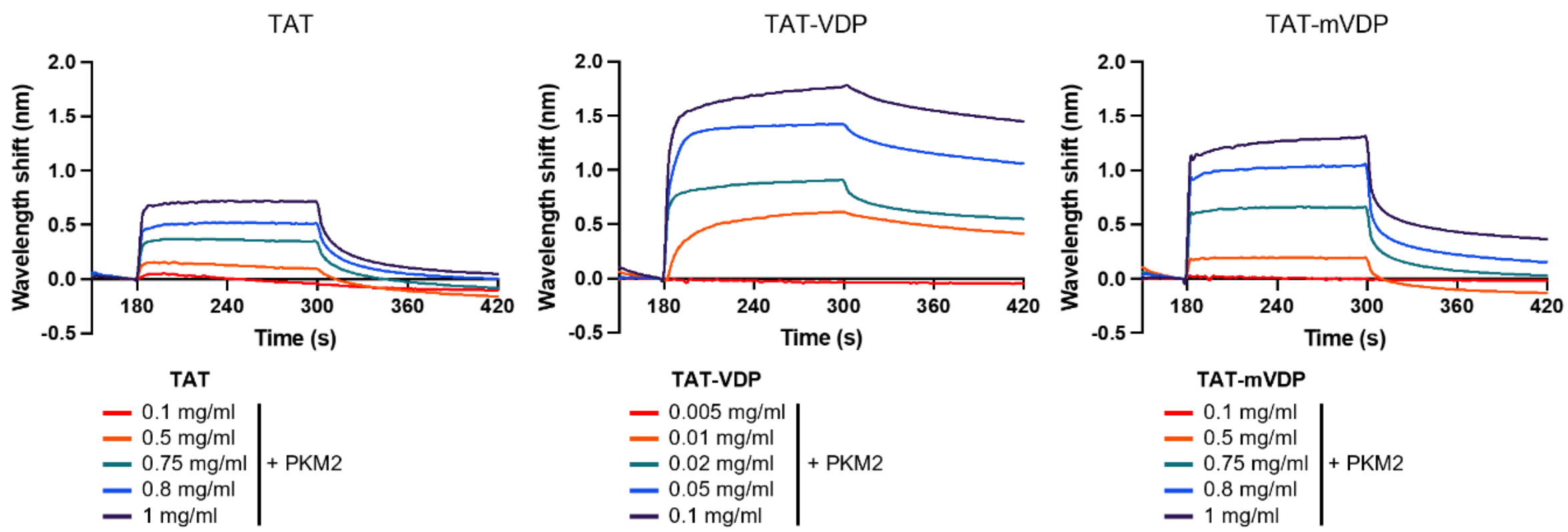
TAT
TAT-VDP
TAT-mVDP
Wavelength shift (nm)
2.0
1.5
1.0
0.5
0.0
-0.5
180
240
300
360
420
Time (s)
TAT
0.1 mg/ml
0.5 mg/ml
0.75 mg/ml
0.8 mg/ml
1 mg/ml
+ PKM2
TAT-VDP
0.005 mg/ml
0.01 mg/ml
0.02 mg/ml
0.05 mg/ml
0.1 mg/ml
+ PKM2
TAT-mVDP
0.1 mg/ml
0.5 mg/ml
0.75 mg/ml
0.8 mg/ml
1 mg/ml
+ PKM2

i

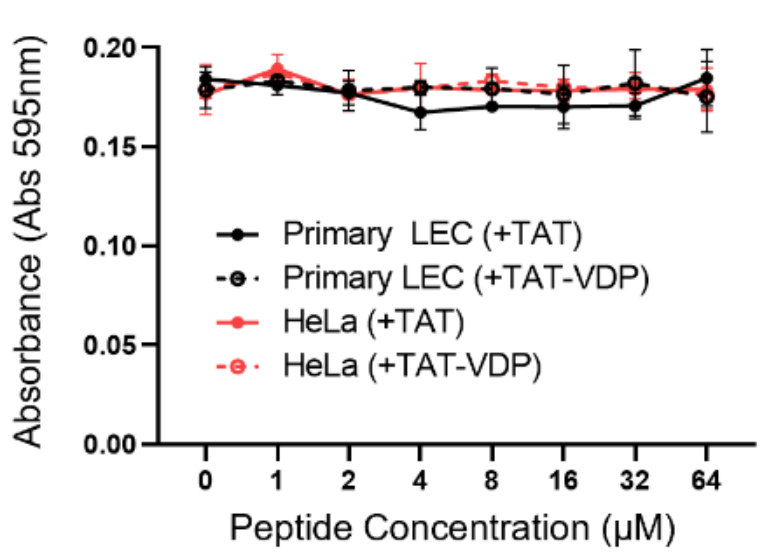
Absorbance (Abs 595nm)
0.20
0.15
0.10
0.05
0.00
Primary LEC (+TAT)
Primary LEC (+TAT-VDP)
HeLa (+TAT)
HeLa (+TAT-VDP)
0
1
2
4
8
16
32
64
Peptide Concentration (μM)

k

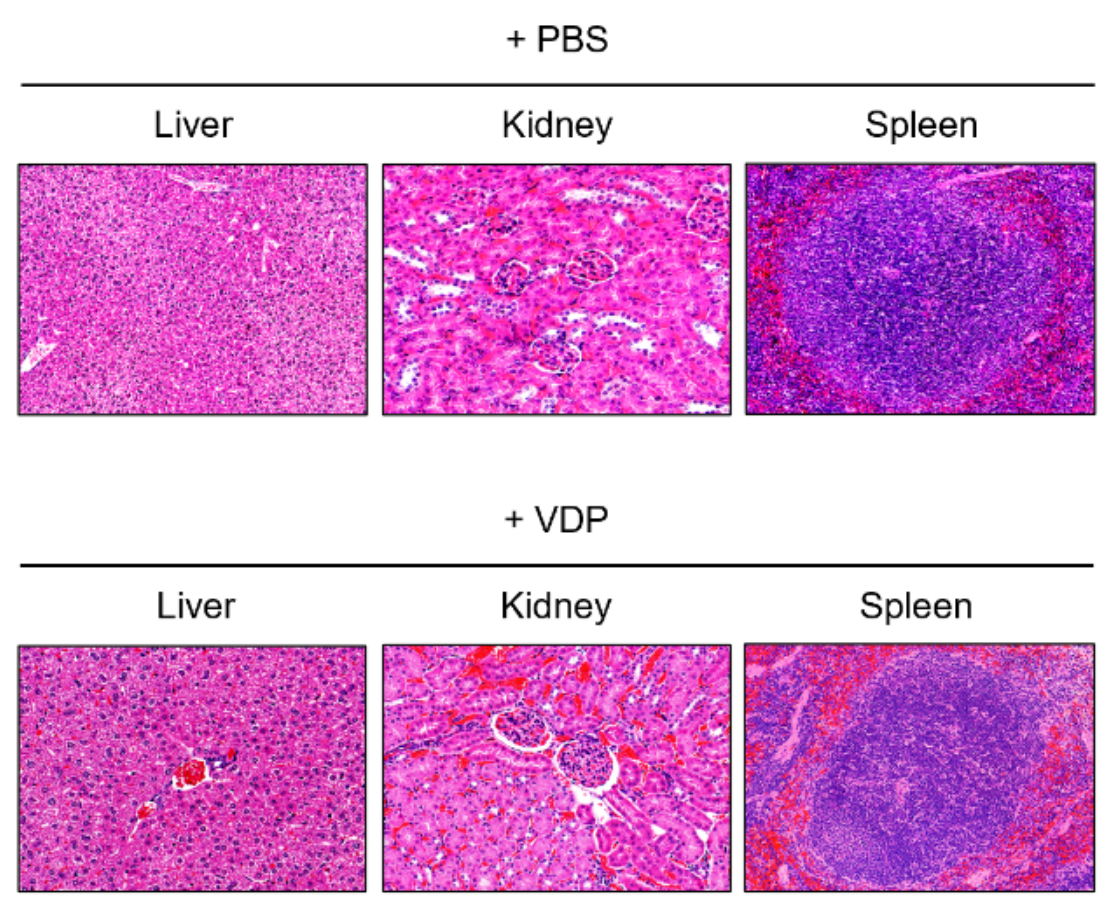
+ PBS
Liver
Kidney
Spleen
+ VDP
Liver
Kidney
Spleen

j

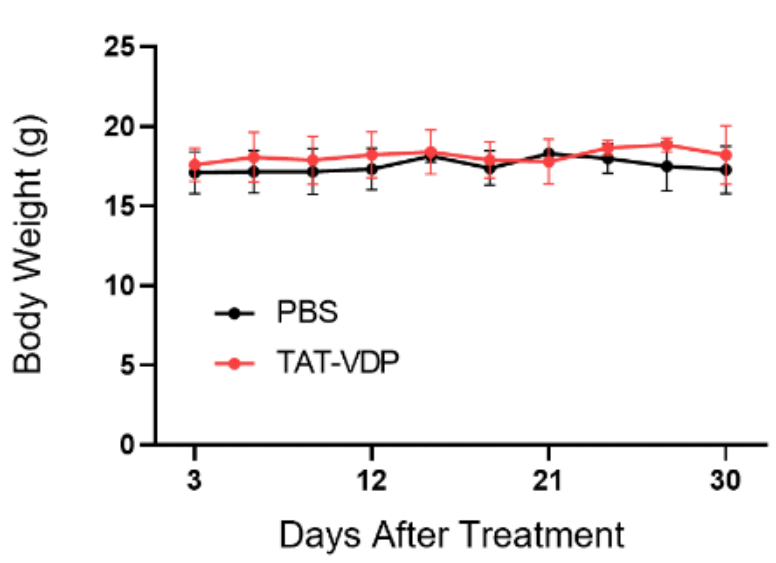
Body Weight (g)
25
20
15
10
5
0
PBS
TAT-VDP
3
12
21
30
Days After Treatment

Supplementary Fig. 5 a 293T cells were transfected with PKM2 and indicated vIRF3 mutant constructs and proceeded to GST-pull down. The pull-down samples were IB with anti-HA antibody. **b** The binding competition assay was performed by co-transfection of vIRF3, PKM2 and vIRF3 peptide region (250-260 a.a.). The lysate was immunoprecipitated with anti-HA antibody followed by IB with anti-myc and anti-GST antibody. **c** LEC-E6/E7-V5-vIRF3 cell line was treated with indicated concentration of TAT or TAT-VDP peptide. The pyruvate kinase activity was measured for each sample 24 hours after peptide treatment. **d** Ribbon diagrams of AFM predicted models of the VDP-mPKM2 complex. (Green: model 1, Yellow: model 2, Red: model 3, Magenta: model 4, Orange: model 5) **e** Ribbon diagrams of VDP bound to the mPKM2 obtained after 50 ns of MD simulations of the complex in two different orientations. Residues involved in the interaction are represented as stick models and labelled (Cyan: PKM2 chain A, Red: VDP). **f** Secondary structure representation of PKM2 monomer. (Red: beta strands, Green: alpha helices). Adapted from figures generated by PDBsum. **g** RMSD plot of VDP throughout the simulation time of VDP-mPKM2 complex. **h** BLI kinetic assay measuring the binding affinity of PKM2 and TAT, TAT-VDP, TAT-mVDP. The curves were globally fitted in a 1:1 mode of interaction. TAT-VDP was found to bind tightly to PKM2 with a K_D value of 1.28 μM while the mutant VDP (mVDP) showed 30-fold lower binding affinity. **i** Cytotoxicity evaluation of TAT and TAT-VDP peptides in primary LEC and HeLa cells. Cells were treated with increasing concentrations of peptides (1-64 μM), and cell viability was assessed using an MTT assay. **j** Changes in body weight of mice following treatment with PBS or TAT-VDP. Body weights were monitored throughout the experimental period. **k** Histological evaluation of major organs following treatment with PBS or TAT-VDP. Representative hematoxylin and eosin (H&E)-stained sections of the liver, kidney, and spleen are shown.

Supplementary Table 1. Interface energy of VDP-mPKM2 in the AFM predicted models.

AFM predicted model	Interface energy (REU)
1	39.396
2	88.275
3	29.306
4	149.048
5	479.29
